# Harmonizing government responses to the COVID-19 pandemic

**DOI:** 10.1038/s41597-023-02881-x

**Published:** 2024-02-14

**Authors:** Cindy Cheng, Luca Messerschmidt, Isaac Bravo, Marco Waldbauer, Rohan Bhavikatti, Caress Schenk, Vanja Grujic, Tim Model, Robert Kubinec, Joan Barceló

**Affiliations:** 1 Hochschule für Politik, Technical University of Munich, Richard-Wagner Str. 1, Munich, 80333 Bavaria Germany; 2 Independent Researcher, Sydney, Australia; 3 School of Humanities and Social Sciences, Nazarbayev University, Kabanbay Batyr Ave., 53, Astana, 010000 Kazakhstan; 4 Faculty of Law, University of Pernambuco, Praça Adolfo Cirne, Recife, 50050-060 Brazil; 5 iSpot, 15831 NE 8th Str #100, Bellevue, 98008 Washington USA; 6 Division of Social Science, New York University Abu Dhabi, Social Science Building (A5), Abu Dhabi, 129188 United Arab Emirates

**Keywords:** Public health, Research data, Government

## Abstract

Public health and safety measures (PHSM) made in response to the COVID-19 pandemic have been singular, rapid, and profuse compared to the content, speed, and volume of normal policy-making. Not only can they have a profound effect on the spread of the disease, but they may also have multitudinous secondary effects, in both the social and natural worlds. Unfortunately, despite the best efforts by numerous research groups, existing data on COVID-19 PHSM only partially captures their full geographical scale and policy scope for any significant duration of time. This paper introduces our effort to harmonize data from the eight largest such efforts for policies made before September 21, 2021 into the taxonomy developed by the CoronaNet Research Project in order to respond to the need for comprehensive, high quality COVID-19 data. In doing so, we present a comprehensive comparative analysis of existing data from different COVID-19 PHSM datasets, introduce our novel methodology for harmonizing COVID-19 PHSM data, and provide a clear-eyed assessment of the pros and cons of our efforts.

## Introduction

From lockdowns to travel bans, government responses to the SARS CoV-2 virus radically affect virtually every dimension of society, from how governments govern to how businesses operate and how citizens live their lives. Comprehensive, high quality and timely access to COVID-19 public health and social measures (PHSM) data is thus crucial for understanding not only what these responses are, but the scale, scope and duration of their effect on various policy areas. These include e.g. the economy, the environment and society, to say nothing of their ostensible function of reducing the spread of the virus itself. Though dozens of research groups have documented COVID-19 PHSM, these individual data tracking efforts provide only an incomplete portrait of COVID-19 PHSM, with many having stopped their efforts entirely, often due to funding constraints^1^.

This article shows how data harmonization, the process of making comparable and compatible conceptually similar data, can create a more comprehensive dataset of COVID-19 PHSM relative to any single dataset alone. Specifically, this article introduces a novel, rigorous methodology for harmonizing COVID-19 PHSM data from December 31, 2019 until September 21, 2021 for the 8 largest existing PHSM tracking efforts:

- ACAPS COVID-19 Government Measures (ACAPS)^2^
- COVID Analysis and Mapping of Policies (COVID AMP)^3,4^
- Canadian Dataset of COVID-19 Interventions (CIHI)^5^
- CoronaNet Research Project COVID-19 Government Response Event Dataset (CoronaNet)^6,7^
- Johns Hopkins Health Intervention Tracking for COVID-19 (HIT-COVID)^8,9^
- Oxford COVID-19 Government Response Tracker (OxCGRT)^10^
- World Health Organization EURO (WHO EURO) dataset on COVID-19 policies (retrieved from the WHO Public Health and Safety Measures (WHO PHSM)^11^)
- US Center for Disease Control (WHO CDC) dataset on COVID-19 policies (retrieved from the WHO Public Health and Safety Measures (WHO PHSM)^11^)

With the help of 500+ research assistants around the world, these datasets are harmonized into the CoronaNet taxonomy to provide a fuller picture of government responses to the pandemic. This amounts to harmonizing around 150,000 observations external to the CoronaNet dataset, which at the time of writing itself has more than 180,000 observations. Not only can harmonizing these different datasets provide a more accurate and complete basis for understanding the drivers and effects of this pandemic, it can also ensure that the data collected by trackers that have stopped their work are not lost and that the original sources underlying this data are preserved to aid future research.

The value of this work is multifaceted. In terms of its value for aiding understanding of COVID-19 PHSM, our discussion of the various challenges faced in harmonizing these 8 datasets not only illuminates difficulties in the data harmonization process but also provides rich detail as to the relative strengths and weaknesses among different datasets. Such a discussion has thus far been missing in the literature and can help researchers adjudicate which dataset best meets their research needs. Initiating this discussion also makes transparent the difficulties in collecting PHSM data more generally, which, to our knowledge, has not been documented in such extensive and comparative detail before. Meanwhile, with respect to its value to COVID-19 research, our ongoing data harmonization efforts represents an enormous improvement over what was previously available from any one dataset alone. While any improvement to PHSM data coverage across time or government jurisdictions can provide a more robust and accurate basis for forwarding research on the drivers and effects of the pandemic, harmonizing data from the 8 largest such existing datasets ensures that our resulting dataset will be able to provide the most comprehensive and detailed information about COVID-19 PHSM possible. Finally, from a methodological perspective, our PHSM harmonization effort can serve as a guide for the harmonization of other datasets, especially in the social sciences. That is, in contrast to virtually all other harmonization efforts we could identify^12^, ours is largely implemented manually, providing us with an unusually intimate knowledge of data harmonization at the level of the individual observation.

In the results section, we provide readers with an overview of our data harmonization efforts from a variety of different angles. We begin from a forward-looking perspective by providing readers insight into the challenges we faced in harmonizing incomplete and dirty data which often suffered from missing sources in order to provide context for the methodological steps we employed to pursue data harmonization. Interested readers can find a fuller accounting of our methodology in the methodology section. The results section ends with a backward-looking perspective by providing an assessment of different aspects of our harmonization efforts, including e.g. the likely gains and limitations of our efforts. Overall, our data harmonization efforts significantly outperform the only other effort we are aware of to harmonize COVID-19 PHSM data, the WHO PHSM effort^11^ (see Supplementary Information, section 3). We conclude with a discussion of how data harmonization illuminates complexities in the data generation process.

## Results

Choosing which datasets to harmonize is one of the most significant decisions a researcher must make when harmonizing data because the particularities of any given dataset can have substantial downstream effects on how they can be made to fit together. Given this, we start this section by outlining our rationale for choosing to harmonize the particular datasets that we do. Having established this basis, we then dive into the the challenges of harmonizing complex, dirty, and incomplete COVID-19 PHSM data. We then briefly show how our methodology can address these challenges (see our methodology section for more information). We end this section by providing an assessment of our efforts to harmonize COVID-19 PHSM data, the criteria for which is based on separate guidance we developed on the topic of data harmonization more generally^12^.

### Which datasets to harmonize?

In adjudicating which COVID-19 PHSM datasets to harmonize, we weighed the potential benefits of data harmonization among a number of different dimensions, including the:

- Geographical coverage
- Temporal coverage
- Volume of data collected
- Relative similarity of policy taxonomies to the CoronaNet taxonomy
- Relative capacity of external dataset partners for collaboration

As can be seen in Supplementary Table 1, we identified more than 20 datasets which could potentially be harmonized into the CoronaNet taxonomy. We ultimately chose datasets to harmonize that (i) aspired to world-wide geographic coverage with (ii) at least ten thousand observations in each dataset and were (iii) based on original coding of sources (as opposed to recoding of existing sources). Datasets that fit this criterion were ACAPS, COVID AMP, HIT-COVID, OxCGRT, and the Complexity Science Hub COVID-19 Control Strategies List (CCCSL)^13,14^ (note, we started but did not finish harmonizing CCCSL data, see below for more information).

One clear exception to this criteria was the inclusion of the CIHI dataset, which focuses on Canadian policies and had fewer than ten thousand policies. We decided to include the CIHI dataset for consideration because i) it already formed a substantial part of subnational data collection for other data collection efforts, including the OxCGRT dataset and ii) of substantial cooperation and access to researchers with expertise in both the CoronaNet and CIHI taxonomies. Similarly, though the WHO EURO dataset aims for a regional, rather than a world-wide focus, given that (i) it is part of the WHO PHSM dataset, which we compare our efforts to in Section 3 of our Supplementary Information (ii) CoronaNet is partially supported by the EU Commission for its EU data collection, we decided to include it for harmonization. Because the WHO CDC dataset follows the same taxonomy as the WHO EURO dataset and also contains a substantial number of policies, it was also included for harmonization.

September 2021 was chosen as the cutoff date given our available resources and because most data tracking efforts had stopped or significantly slowed their data collection by this date except for OxCGRT, CIHI and WHO EURO (OxCGRT stopped in 2023 while the latter two stopped in 2022). Should more resources become available we will expand our efforts to harmonize records for these datasets beyond this date.

### Challenges of Data harmonization

Harmonizing data is rarely straightforward, but harmonizing COVID-19 PHSM data was particularly challenging because standards which researchers usually abide by before releasing data were not observed due to the emergency nature of the pandemic. For one, to the extent that researchers generate event-based datasets, they normally concern past events, not ongoing ones. Indeed a given event must run its course in order for researchers to both i) conceptualize the event being captured into a structured and logically organized taxonomy ii) estimate the amount of work needed in order to build a dataset based on that taxonomy. For another, because dirty data can significantly bias subsequent research findings, researchers often err on the side of caution by spending substantial additional time rigorously cleaning and validating the data before release. Researchers also have personal incentives to delay releasing data given that i) they generally wish to be the first to conduct analyses on data that they themselves have collected and ii) unclean datasets can significantly negatively affect professional reputations. Meanwhile, to promote replicability and transparency about the data generating process, documentation of original sources and coding decisions are often extensive. Due to the pandemic, however, PHSM data exceptionally were:

- Collected based on taxonomies that were developed inferentially from research group to research group while the COVID-19 pandemic was still ongoing.
- Released without extensive cleaning.
- Inconsistently preserved with regards to original raw sources.
- Absent regular documentation on changes to taxonomies or data collection methods.

There were a number of research-based reasons to prioritize speed over rigor. Not only did launching data collection during rather than after the pandemic help jump start early COVID-19 research, in many cases it was critical to document these policies in as close to real time as possible because primary sources on the pandemic can and have disappeared from the Internet over time. Meanwhile, though many COVID-19 trackers surely would have continued to improve their data quality, unfortunately many stopped their efforts because of lack of funding support. Our efforts to harmonize this external data into the CoronaNet dataset thus not only ensures that their substantial contributions can live on, but are also improved insofar as any errors in the data or discrepancies between datasets have a higher chance of being identified and resolved before being harmonized. This job is made more difficult however, because many trackers did not have rigorous guidelines for preserving raw sources. In what follows, we expand upon how each of these pandemic-related challenges have affected our data harmonization efforts and subsequent methodological decisions.

### The challenge of harmonizing different taxonomies

Different conceptualizations of what ultimately ‘counts’ as PHSM data lie at the root of different taxonomic approaches to collecting such data. While one benefit of independently developing taxonomies is that it encourages greater flexibility and adaptability in conceptualizing COVID-19 PHSM while simultaneously validating common themes that independently appear across taxonomies, it also makes reconciling the differences among them more challenging. A particular challenge with our data harmonization efforts is that the CoronaNet taxonomy on the whole captures more policy dimensions than other datasets do. While this means that our data harmonization efforts will yield much more fine-grained information on a given policy than would be available in its original form, mapping a simpler taxonomy into a more complex taxonomy is also a much more challenging task compared to vice-versa. In what follows, we discuss what challenges we faced when mapping taxonomies for COVID-19 policy types in particular as well as for other important dimensions of COVID-19 policies.

There are at least four broad issues to consider when mapping the substance of different COVID-19 policies: (i) when taxonomies use the same or similar language to describe a policy but ultimately conceptualize them differently (ii) when taxonomies have the same or similar conceptual understandings of a given event but differ in how they record the data structurally (iii) when taxonomies have similar but ultimately different conceptual understandings of a given event and (iv) when taxonomies capture and conceptualize different events. We elaborate with examples for each of these issues in what follows:

Different datasets can often use similar language to describe conceptually different phenomena. An example of why it is important to be sensitive to these semantic details can be seen with regards to the term ‘restrictions on internal movement.’ While all datasets that use this terminology understand this to entail policies that restrict movement, some have different understandings of the term ‘internal.’ For instance, because OxCGRT generally codes policies from the perspective of the country (note OxCGRT does document subnational data for a select number of countries: the United States, Canada and China. Further note that although OxCGRT also collects subnational data for Brazil, in this case, it appears that their subnational Brazilian data is also coded at the level of the country), their ‘C7_Restrictions on internal movement’ indicator captures any restriction of movement within a country. Meanwhile, because CoronaNet codes policies from the perspective of the initiating government, its ‘Internal Border Restrictions’ policy type captures policies that restrict movement within the jurisdiction of a given initiating government while policies that restrict movement outside a given jurisdiction are coded as ‘External Border Restrictions.’ As such, if the state of California restricts its citizens from leaving the state, this would be captured in OxCGRT’s ‘C7_Restrictions on internal movement’ indicator but would be coded as an ‘External Border Restriction’, not an ‘Internal Border Restriction’ using the CoronaNet taxonomy. Parsing out these differences can only be automated to a limited extent, especially if the taxonomies being mapped simply do not make the same distinctions.

Meanwhile an example of how different datasets implemented different taxonomic structures to capture a similar conceptual understanding of a policy is how they captured policies related to older adults. OxCGRT organized its taxonomy by creating an ordinal variable with its “H8_Protection of elderly people” index. Specifically, this index records “policies for protecting elderly people (as defined locally) in Long Term Care Facilities (LTCFs) and/or the community and home setting” on an ordinal scale (it takes on a value of 0 if no measures are in place, 1 if restrictions are recommended, 2 if some restrictions are implemented and 3 if extensive restrictions are implemented; see the OxCGRT codebook^10^ for further details). In contrast, the CoronaNet and COVID AMP taxonomies document policies toward older adults not in its policy type variable but in a separate variable which records the demographic targets of a given policy (in CoronaNet, this is the ‘target_who_gen’ variable while in COVID AMP this is the ‘policy_subtarget’ variable). Both datasets record whether a policy is targeted toward ‘People in nursing homes/long term care facilities.’ CoronaNet additionally makes it possible to document whether a policy is targeted toward ‘People of a certain age’ (where the ages are captured separately in a text entry) or ‘People with certain health conditions’ (where the health conditions are captured separately in a text entry) while COVID AMP additionally makes it possible to document whether a policy is targeted toward ‘Older adults/individuals with underlying medical conditions.’ When mapping different taxonomies, these differences in taxonomic structure must additionally be taken into account.

Furthermore, taxonomies may capture similar, yet conceptually still quite distinct events which makes one-to-one matching between datasets difficult, if not impossible. For instance, the CIHI taxonomy’s policy type of ‘Travel-restrictions’ does not make any distinctions between restrictions made within or outside of a given government’s borders. Meanwhile, to revisit the example of policies related to older adults, by developing a ‘nursing homes’ category, HIT-COVID taxonomy targets not older adults per se, but the institutional settings in which they are likely to be the most vulnerable. The WHO PHSM dataset meanwhile generalizes this idea in its policy category of ‘Measures taken to reduce spread of COVID-19 in settings where populations reside in groups or are restrained or limited in movement or autonomy (e.g., some longer-term health care settings, seniors’ residences, shelters, prisons).’ This taxonomy implicitly suggests that it may be prudent to investigate not only the effects of policies on older adults but for all those with limited mobility at the expense of easily extractable information on older adults in particular. Cases such as these are perhaps the most difficult to resolve as it is impossible to directly map distinctions that one taxonomy makes into other taxonomies where no such distinctions are made.

Finally, while all datasets generically sought to capture policies governments made in response to COVID-19, different datasets focused on different policy areas. For instance, virtually all external datasets have separate policy categories to capture economic or financial policies (e.g. government support of small businesses) while such policies are not systematically captured in the CoronaNet taxonomy. In these cases, such policies are thus simply not mappable.

The fact that different projects undertook such a variety of approaches in capturing PHSM policies also underscores the idea that there is no one correct taxonomy for capturing such policies; each has its own pros and cons. For instance, aggregating all policies towards older adults in one indicator as OxCGRT does facilitates research on how the pandemic affects older adults but makes it difficult to easily compare the effect of the pandemic on other vulnerable populations. Meanwhile though the CoronaNet and COVID AMP approach allows more flexibility in what kind of policies toward older adults can be captured, it also lacks the cohesiveness the OxCGRT indicator for older adults enjoys. With regards to data harmonization meanwhile, the sheer variety of approaches substantially increases the challenge of transforming PHSM data to adhere to one taxonomy.

Indeed, despite a strong partnership with CCCSL, we opted not to harmonize data from the CCCSL dataset because of these taxonomic challenges. We found CCCSL’s structure and semantics were too different from CoronaNet’s, such that we estimated we would ultimately only be able to use less than half of CCCSL’s observations. To illustrate by example, an observation with the CCCSL id of 4547 notes in its description that ‘Ski holiday returns should take special care.’ Such an observation would not be considered a policy in the CoronaNet taxonomy because it is does not provide specific enough information about what is meant by ‘special care’ and the link for the original source of this observation is dead. While many observations do contain high quality information and descriptions, a substantial number do not contain any or only very minimal descriptive information. Combined with the difficulty in accessing original sources, we decided the relative effort required to consistently map the remaining observations into the CoronaNet taxonomy would be too high, especially considering that we are also harmonizing similar data from 7 other datasets.

So far we have only discussed the challenge of mapping taxonomies specific to policy types. However all datasets also capture additional important contextual information for understanding, analyzing and comparing government COVID-19 policies. In Table 1 below, we show the variety of approaches different datasets undertook to capture some of the most important of these dimensions including: the data structure (Structure), whether a given dataset captures end dates (End Dates?), has a protocol for capturing and linking updates of a policy to its original policy (Updates?), has a standardized method for documenting policies occurring at the provincial ISO-2 level (Location standardized at ISO-2 level?), captures information about the geographic target of a policy (Geog. Target?) or captures information about the demographic target of a given policy (Demog. Target?).

**Table 1:** Comparison of dimensions captured across different datasets.

Dataset	Structure	End Dates?	Updates?	Location standardized at ISO-2 level?	Geog. Target?	Demog. Target?
CoronaNet	Event data	Yes	Yes	Yes	Yes	Yes
ACAPS	Event data	No	No	No	No	No
CIHI	Event data	Extractable through description text field	No	Yes	Yes	No
COVID AMP	Event data	Yes	Yes	Yes	Yes	Yes
HIT-COVID	Event data	No	Yes	Yes	No	No
OxCGRT	Panel data	Yes	NA	Yes	No	No
WHO (CDC and EURO)	Event data	Yes	Yes	No	No	No

As Table 1 shows, while most external datasets are formatted in event dataset format which facilitates comparability across these datasets, OxCGRT data is available only in panel format, which presents unique challenges. With regards to the data structure, in order to facilitate data harmonization, the OxCGRT data must be reformatted to an event format (see the Supplementary Information, Section 2, to access the taxonomy map). However, the panel structure also has knock-on effects on how other policy dimensions are captured, which we discuss in greater detail later in this section.

Datasets also differ with regards to how they capture the timing of a policy. Although knowing the duration of a policy is crucial for understanding its subsequent impact, if any, neither ACAPS and nor HIT-COVID systematically captured information about policy end dates. Though CIHI did make this data available through its textual description, it was not available as an individual field and had to be separately extracted. When harmonizing data from these datasets then, additional work must be done to provide information on end dates.

Relatedly, datasets have also taken inconsistent approaches to capturing policy updates, if at all. Taxonomies that capture such updates are arguably better equipped to capture the messiness and uncertainty of the COVID-19 policy making process (e.g. policy makers often lengthen or shorten the timing of a given policy in response to changing COVID-19 conditions). ACAPS and CIHI however do not separately capture and link policy updates to the original policy. Meanwhile, OxCGRT’s inability to capture information on how policies may be linked is largely due to its panel dataset structure. In contrast, though both the CoronaNet and COVID AMP taxonomies have rules for linking policies together, these differ across datasets. Specifically, CoronaNet links policies together if there are any changes to the original policy’s time duration (e.g. extended or reduced over time), quantitative amount (e.g. number of quarantine days), directionality of the policy (e.g. whether a policy targets outbound or inbound travel), travel mechanisms (e.g. whether a policy targets air or land travel), compliance (e.g. whether a policy is recommended or mandatory), or enforcer (e.g. which ministry is responsible for implementation). COVID AMP meanwhile, has separate fields to document i) whether an original policy was extended over time (see the ‘prior row id’ in the COVID AMP dataset) or ii) whether a given policy implemented at the local level has a relationship with a higher level of government (see the ‘Parent policy number’ field in the COVID AMP dataset).

While all datasets use a standardized taxonomy for documenting country level information about where a policy originated from, some datasets did not use a standardized taxonomy for capturing this information at the subnational ISO-2 level, in particular ACAPS and the WHO. Even when the taxonomy was standardized within a given dataset, different datasets used slightly different taxonomies at both the country and subnational levels which also necessitates further reconciliation and standardization across datasets .

Of all the datasets processed for data harmonization, only the CoronaNet and COVID AMP datasets capture information on both the particular geographic (e.g. country, province, city) and demographic targets (e.g. general population, asylum seekers) of a given policy. To the extent that other datasets also capture this information, it is either very broad or not standardized enough. For instance, though the various indicators in the OxCGRT data capture whether a policy overall applies to the general population or a targeted population, no further information about the specific targets is provided. Meanwhile, the WHO PHSM dataset does have a separate field which documents demographic targets but these entries are not standardized resulting in more than 5900 unique categories, many of which have typos (see Supplementary Information, Section 3, for more). It is thus impossible to use them for analysis without substantial additional cleaning and harmonization.

All in all, harmonizing different datasets can be challenging when considering only two taxonomies, much less eight. This is true not only with regards to taxonomies specific to the substance of COVID-19 policies themselves but also with respect to additional policy dimensions like policy timing and targets.

### The challenge of harmonizing dirty data

Dirty data refers to data that is miscoded with reference to a given taxonomy. In our investigation of the cleanliness of different datasets, we distinguish between policies that are (i) inaccurately coded relative to a given taxonomy or (ii) incomplete or missing. Harmonizing dirty data would be challenging even if taxonomies across datasets were the same; these problems are only compounded when taxonomies are different. Unfortunately, because of the pandemic emergency, all datasets considered here suffer from problems with dirty data.

For instance, although within the ACAPS taxonomy, all policies related to curfews should theoretically be coded as ‘Movement Restrictions’ and ‘Curfew’ in their ‘category’ and ‘measure’ fields respectively, text analysis of the descriptions accompanying these observations suggests that curfew policies were mistakenly coded into at least 8 other policy categories. For example policies relevant to curfews which likely should have been coded as ‘Movement Restrictions – Curfew’ were also found as being coded under other categories like: ‘Lockdown – Partial Lockdown’, ‘Movement restrictions – Surveillance and monitoring’, and ‘Movement Restrictions – Domestic travel restrictions.’ Although admittedly, the aforementioned categories are conceptually quite close to the concept of curfews, curfew measures were also found to be coded under categories that are arguably quite father afield like: ‘State of Emergency’, ‘Movement Restrictions – Border closures’, ‘Public health measures – Isolation and quarantine policies’, ‘Governance and socio-economic measures – Emergency administrative structures activated or established’ and ‘Governance and socio-economic measures - Military deployment.’

Data can also be dirty for other important policy dimensions, e.g. the start dates of a given policies. Many governments maintain websites where they note the most current policies without detailed information as to when the policy started or will end. To draw an example of a government source for Latvian polices (see https://web.archive.org/web/20210621102402/https://covid19.gov.lv/en/support-society/how-behave-safely/covid-19-control-measures), the date the information on policies was updated was June 21, 2021 but the policies themselves were not necessarily implemented on that day. Further research would be needed to triangulate the start date of a given policy listed on this website. Similarly, in some cases, coders will simply record the date that they accessed the policy as the start date as opposed to the true start date. That these types of issues were found across all datasets is no surprise given the unusual circumstances that such data are collected and released. Nevertheless, they can pose immense challenges; blindly harmonizing such data risks compounding the original errors in the data.

While it is difficult to quantify the relative cleanliness of different datasets (and thus, how much of an issue it poses to data harmonization), we provide some sense of their relative data quality with regards to the quality of their textual descriptions in Table 2 below. Good textual descriptions of a given policy are crucial for helping users understand what policies a given dataset is actually documenting and organizing. Table 2 shows how informative these descriptions are by counting, per dataset, the average number of characters each description has (Description Length (Average)), how many descriptions have less than 50 characters (Descriptions with less than 50 characters (Total)) and how many observations have no descriptions at all (Missing Descriptions (Total)).

**Table 2:** Assessment of Textual Descriptions.

	Description Length (Average)	Descriptions with less than 50 characters (Total)	Missing Descriptions (Total)	Description Standardized?	Example of Average Description
CoronaNet	359	266	0	Yes	On 2 April 2020, the Australian Capital Territory government announced the construction of a temporary COVID-19 Emergency Department through a partnership with local healthcare provider Aspen Medical. The package also provides funding to our hospitals to purchase more equipment and more personal protective equipment for our nurses and doctors
ACAPS	172	2147	118	No	IKR extended until at least 23.4.2020 All movements in the Kurdistan Region are banned between the hours of midnight and 0600, except for security officials and ambulances.
WHO CDC	537	126	47	No	Curfew extented. Guatemala s President on 7 June announced he extended curfew and measures imposed to contain the coronavirus pandemic for one more week. The partial curfew, in force from 22 March, will continue between 18H00 and 05H00 starting 8 June, and transit between provinces remains prohibited.Since March, measures such as the suspension of public transport and classes have been in force in Guatemala. Social, religious, sporting and cultural activities are also prohibited. Air, sea and land borders are closed to foreigners.
CIHI	254	0	0	Yes	Who: Government of Yukon What: Updated school health and safety guidelines for K-12 to reduce the requirement for 2-metre distancing between students in the classroom and to make masks mandatory in common areas outside of the classroom. Effective until
COVID AMP	227	950	0	No	Extension: Indoor events with over 500 spectators/ attendees cannot exceed 50% of the venue’s room’s capacity. Indoor events attended by non-students must adhere to social distancing requirements and face covering requirements
WHO EURO	297	509	662	No	Extension through 1 March 2021 Taking into account the analysis of the current situation and the epidemiological situation in the UK, Italy, Germany, Denmark, Austria, Australia, the Netherlands and South Africa, extension of suspension of international flights entry, exit and transit flights .
HIT-COVID	230	635	1622	No	In case the person has any history in the last 14 days and the person is symptomatic as per the case definition of COVID 19, the person must be isolated in a hospital as per protocol and will be tested for COVID19 as per protocol.
OxCGRT	329	4265	0	No	The two week self-quarantine has been lifted for those traveling to New Hampshire from surrounding New England States (Maine, Vermont, Massachusetts, Connecticut, Rhode Island). Those traveling to New Hampshire from non-New England states for an extended period of time are still asked to self-quarantine for a two-week period.

Generally, descriptions with less than 50 characters contain only limited information about a given policy. The following examples from each dataset shows that often missing from these shorter descriptions are dates, places and enforcers of policies and even sometimes the nature of the policy itself: “Albania banned all flights to and from the UK.” (CoronaNet); “Blida extended until at least 19. april 2020” (ACAPS); “Lockdown extended. Lockdown extended” (WHO CDC); “The state of emergency in WA has been extended.” (COVID AMP); “Delay of international flights have been extended” (EURO); “Extends school closures until March 16” (HIT-COVID); “orders extended until April 30” (OxCGRT). The table shows that textual descriptions from the ACAPS dataset have on average the least number of characters compared to others, with more than two thousand having descriptions of less than 50 characters and more than 100 having no description at all. While OxCGRT has the third highest average description length, it also has the most number of descriptions with less than 50 characters. Meanwhile HIT-COVID has the most number of policies without any description, at more than 1600.

With regards to the content of the descriptions, only the CoronaNet and CIHI databases appear to standardize what should be included in this textual description (see ‘Description Standardized?’ column in Table 2). Coders for CoronaNet are instructed to include the following information in their textual descriptions: (i) the name of the country from which a policy originates (ii) the date the policy is supposed to take effect (iii) information about the ‘type’ of policy (iii) if applicable, the country or region that a policy is targeted toward (iv) if applicable, the type of people or resources a policy is targeted toward and (vi) if applicable, when a policy is slated to end. The CIHI descriptions take a regularized format in which the government initiating the policy is clearly specified, the policy type is described and the end date of a given policy is recorded if applicable. With regards to the other datasets, we were unable to find any documentation that suggested that text descriptions should follow a standardized format nor were we able to find evidence of any by reading through a sample of the text descriptions themselves.

For each dataset, we randomly selected one description that accorded to the average description length for that dataset to illustrate what kind of information could be gleaned from them in the ‘Example of Average Description’ column in Table 2. These descriptions suggest that while the CoronaNet and CIHI descriptions include information about the date the policy is enacted and the policy initiator, this information is not always reliably made available for descriptions from other datasets. While this information is generally also subsequently captured in separate variable fields, having detailed textual descriptions are important for helping to adjudicate whether the subsequent coding of these separate policy dimensions is accurate or not.

While it would be useful to have a similar quality assessment for other variables of each dataset, as far as we know, only the CoronaNet dataset provides an empirical assessment of the quality of its data. CoronaNet implements a multiple validation scheme in which it samples 10% of its raw sources for three independent coders to separately code. If 2 out of 3 of the coders document a policy in the same way, then it is still considered valid. Though data validation is still ongoing, preliminary data from Table 3 suggests that inter-coder reliability is around 80% for how its policy type variable is coded, a level which is generally accepted to be indicative of high inter coder reliability^15–17^ An exception to the generally high validity of the policy type variable is the relatively poor coder interreliability for the ‘Health Testing’ and ‘Health Monitoring’ categories. This is likely related to changes in the CoronaNet taxonomy, which while important to make to better adapt to the changing policy-making environment, also increases the dirtiness of the data. A full accounting of taxonomy changes can be found by accessing the CoronaNet Data Availability Sheet https://docs.google.com/spreadsheets/d/1FJqssZZqjQcA-jZhRnC_Av9rlii3abG8r7utBeuzTEQ/editgid=1284601862.

**Table 3:** Inter-Coder Reliability Measures for Ongoing Data Validation (Round 1).

Policy	(n)	Percentage	Cohen’s
		Agreement	Kappa (k)
Curfew	19	100	1
Hygiene	2	100	1
Declaration of Emergency	54	96.3	0.96
Restrictions of Mass Gathering	92	94.6	0.94
External Border Restrictions	112	94.6	0.94
Closure and Regulation of Schools	48	93.8	0.93
Restriction and Regulation of Businesses	63	90.5	0.9
Lockdown	28	85.7	0.85
Restriction and Regulation of Government Services	63	84.1	0.83
Health Resources	91	83.5	0.82
Quarantine	47	83	0.82
Internal Border Restrictions	22	81.8	0.81
Social Distancing	37	73	0.71
New Task Force, Bureau or Administrative Configuration	22	72.7	0.71
Public Awareness Measures	51	68.6	0.67
Health Testing	17	52.9	0.5
Health Monitoring	13	23.1	0.18
**Summary of Inter-coder Reliability Scores**			
Percentage Agreement	81.7		
Cohen’s Kappa	0.8		
Krippendorrf’s allpha	0.82		

Other external datasets have likely faced similar issues which subsequently affect their data quality although we were unable to locate public documentation of these changes. Note however, if there were any taxonomy changes for OxCGRT or HIT-COVID, they are likely recoverable from their Git repository histories. The closest similar information that other datasets provide on data quality are with regards to their cleaning procedures. More information on the steps other datasets took to ensure data quality can be found in their respective documentation (see: CoronaNet^6^; ACAPS^2^, OxCGRT^10^, HIT-COVID^8^ and the WHO PHSM^18^; no documentation on data quality procedures was found for CIHI). Given that a number of external trackers stopped their data collection efforts as well as the relatively high level of data quality of the CoronaNet data for the dimensions that we have information on, we can cautiously infer that harmonizing external data to the CoronaNet dataset will help improve the quality of the subsequently harmonized data.

Data completeness is also an important factor in a dataset’s overall quality. The more complete a datatset is, the more accurate subsequent analyses based on this data can be. All datasets harmonized here are by definition incomplete given that they made their datasets publicly available while their data collection efforts are ongoing. This issue is compounded by the fact that many datasets have had to stop or substantially slow their data collection efforts, particularly ACAPS, HIT-COVID, WHO CDC and COVID AMP. Because policies often continue past the lifetime of the group collecting the data itself, issues of data incompleteness only grow over time for datasets that stop collecting data. While a full assessment of the completeness of each dataset is not possible (one would need a perfectly complete dataset in order to judge the completeness of other datasets) in Table 4 below, we provide some sense of each dataset’s relative completeness by assessing how many policies lack end dates in terms of the raw data and the harmonized data as well as the average start and end dates of policies and the last submission date of a given policy.

**Table 4:** Assessment of Data Completeness.

Dataset	Missing End Dates (Total Raw)	Missing End Dates (% Raw)	Missing End Dates (% Harmonized)	Start Date (Raw Average)	End Date (Raw Average)	Last Submission Date
CoronaNet	27932	29.0	NA	2020-08-24	2020-12-20	2023-12-01
ACAPS	18699	100	51.72	2020-06-13	NA	2020-12-08
WHO CDC	1384	19.50	42.51	2020-08-16	2020-09-06	2021-12-05^1^
CIHI	3483	82.73	86.73	2020-10-06	2020-11-15	2021-08-12^3^
COVID AMP	4970	18.77	25.47	2020-08-28	2020-10-09	2021-09-21^3^
WHO EURO	2137	15.03	35.96	2020-09-10	2020-09-24	2021-06-21^2^
HIT-COVID	8142	100.00	55.61	2020-04-15	NA	2020-12-15
OxCGRT	2558	3.59	39.06	2020-12-27	2021-01-07	2021-09-21^3^

^1^There is likely some issues with this variable for the WHO CDC data as we retrieved the WHO PHSM data on 2021-09-10 so the last submission date could not have been after this date.

^2^There was only one submission date recorded for the WHO EURO dataset (2020-04-01), however according to the WHO PHSM website, the last update was 2021-06-21.

^3^These datasets do not separately record this information. Instead the date the dataset was retrieved or the self-reported last submission date for the entire dataset was used.

The first column, ‘Missing End Dates (Total Raw)’ shows that on an absolute basis, CoronaNet has by far the greatest number of missing end dates. However this large number is largely a function of the large size of its dataset (see Table 6 for information on size of different datasets). When we turn to the second column ‘Missing End Dates (% Raw)’, which assesses the percentage of end dates missing from the data before it is manually assessed for data harmonization, the table shows that following ACAPS and HIT-COVID which, as previously mentioned, do not collect information on any end dates at all, CIHI has the highest percentage of missing end dates while the OxCGRT data has the lowest percentage. However, this column should be contrasted with the subsequent one, ‘Missing End Dates (% Harmonized)’ (note that the ‘raw’ version of the data presented here corresponds to ‘Step 3’ of the data and the ‘harmonized’ version of the data presented here correspond to ‘Step 5’ of the data. See Table 6 for a precise breakdown of this data and the methodology section for more information as to how the data was processed during this different steps), which shows the prevalence of missing end dates for policies that have been assessed for harmonization. Here we can see that both ACAPS and HIT-COVID have improved in terms of the prevalence of missing data — this suggests that while they themselves did not systematically gather information on end dates, it was possible for research assistants to recover this information from the raw sources that they were based on during the harmonization process.

The difference between what was reported in the raw data and what was assessed during the harmonization process is particularly drastic in the case of OxCGRT data and given that it suggests that the OxCGRT data is of substantially poorer quality than it appears, it deserves some additional attention. In particular, though OxCGRT was originally assessed as having missing end dates for around 3% of the data when based on the raw data, this percentage explodes to nearly 40% during the harmonization process. A likely explanation for this discrepancy is that the OxCGRT data is collected as an ordinal index in a panel form. To take border policies as an example, the OxCGRT index for border closures takes a value of 3 if borders are closed to all countries and a 4 if it is closed to all countries. If country X (i) only bans travel from country A in March 2020 (ii) then only bans travel from country B (lifting the ban for country A) in April 2020, and finally (iii) bans travel all countries in May 2020, it will take on a value of 3 according to the OxCGRT in March and April of 2020 and a value of 4 in May of 2020. However, it is not necessarily the case that the OxCGRT data will accurately record the end date of the travel ban against country A since for the purposes of its index, the same value of 3 is maintained throughout March and April. Note that generally, all of OxCGRT ordinal indexes follow a similar logic insofar as they document whether a restriction applied to all, none or some but do not provide further specifics when the restriction only applies to some. Such lapses in documentation likely explain the comparatively high number of missing end dates for the OxCGRT data.

Overall, when considering the numerous different dimensions of the missing end dates problem, our analysis suggests that COVID AMP and CoronaNet have the highest data quality in terms of having the lowest percentage of data without missing end dates, though COVID AMP’s relatively better performance is likely a function of the smaller size of its dataset. All datasets however, are evaluated as having a more severe problem with missing end dates when they are being assessed for harmonization, rather than in their raw form, with the exception of ACAPS and HIT-COVID which did not systematically collect data on end dates. This discrepancy underscores the challenge in harmonizing dirty data. Note that there is no value given for CoronaNet in terms of the percentage of missing end dates for the harmonized data because the harmonized and raw versions of the dataset are identical for CoronaNet.

Meanwhile, based on the raw version of the data, though the average start date and end dates for all datasets center around the last half of 2020, the earliest average start dates are found in ACAPS, and HIT-COVID, with OxCGRT, CIHI and WHO EURO being relatively farther along and CoronaNet, CDC and COVID AMP in the middle of the pack. Meanwhile WHO CDC and WHO EURO have the earliest average end dates while OxCGRT, CIHI and CoronaNet have the latest average end dates. The last submission date (relative to September 2021 when the data was last retrieved for all datasets except CoronaNet) shows when datasets have stopped or slowed their data collection efforts. Overall then, this table suggests that data harmonization of these 7 other datasets into the CoronaNet dataset may substantially raise the data completeness of the CoronaNet dataset.

As outlined above, all datasets considered in this paper suffer in various degrees from problems of miscoded or missing or incomplete data. However, though dirty data substantially raises the complexity and challenge of accurate data harmonization, the data harmonization process can also improve the quality of such data, which we will discuss in more detail later on.

### The challenge harmonizing data with missing information on original sources

Given both the challenges in harmonizing (i) data coded from multiple different taxonomies as well as (ii) dirty data, it is essential to have access to the original raw source of data of a given policy to harmonize the data accurately. Original sources are necessary to substantiate the content or nuances of a given policy or to resolve any confusion or disagreement about a given coding decision.

In Table 5, we illustrate differences among each dataset in terms of how they make source data available (Source Data) and how many observations do not have any source data attached to it (Missing Links (Total)). The table also shows, relative to external data that has already been assessed for harmonization, the percentage of observations that have been found to be based on sources with dead links for which corroborating information was unable to be found after a good faith effort (Unrecoverable links (Percent of total harmonized)) as well as the percentage of observations which have been found to be based on dead links but for which corroborating information was subsequently recovered (Recovered Links (Percent of total harmonized)).

**Table 5:** Assessment of Raw Sources.

Dataset	Source Data	Missing Links (Total)	Unrecoverable links (Percent of total harmonized)	Recovered Links (Percent of total harmonized)
CoronaNet	URL and PDF links	0	NA	NA
ACAPS	URL links	26	12.80	5.17
WHO CDC	URL links	24	8.30	8.52
CIHI	URL links	0	5.91	1.08
COVID AMP	URL and PDF links	8	2.89	4.06
WHO EURO	URL links	3011	20.70	7.47
HIT-COVID	URL links for early data; URL links and PDF links for later data	14	4.37	4.92
OxCGRT	URL links	1385	6.39	2.90

We find that while all datasets provide reference to the URL links used to code a given policy, only CoronaNet, COVID AMP and HIT-COVID also provide links to static PDFs of raw sources which ensure that this information can continue to be available in the future. Note however, COVID AMP has around 150+ observations which only have a URL link and no PDF link attached to it while early observations entered into the HIT-COVID dataset also only have URL links with no accompanying PDF links. With regards to the extent to which a given observation is missing a URL or PDF link to its raw source, the WHO EURO and OxCGRT datasets have the most number of missing links while this is not an issue for the CoronaNet and CIHI datasets. Meanwhile, based on the amount of external data that has been harmonized thus far, around 10.2% of the external data is based on links that were dead which were not possible to recover corroborating information for. This was a particular problem for the WHO EURO and WHO CDC datasets though not an issue for the CIHI or COVID AMP datasets. Meanwhile around 4.7% of the external dataset assessed for harmonization to date, were based on dead links but for which it was possible to recover corroborating information. Because these data points are recoded using the CoronaNet methodology, PDFs of these recovered links were also uploaded, ensuring that they will continue to be preserved for future records. Observations coded by the WHO EURO database were found to be particularly recoverable. Note that we do not make an assessment for unrecoverable or recovered links for CoronaNet because the CoronaNet methodology ensures that PDFs are always saved (the data is collected via a survey and uploading a PDF is mandatory for a policy response to be considered valid). All told, at least 17% of the external data (3% of the external data have no links, 10.2% of the data are based on links with unrecoverable information and 4.7% of the data are based on links with recoverable information) are based on data with some issues with regards to their original sources, which only increases the challenge of smoothly harmonizing information from different datasets.

### COVID-19 PHSM Data Harmonization methodology overview

**Fig. 1:**
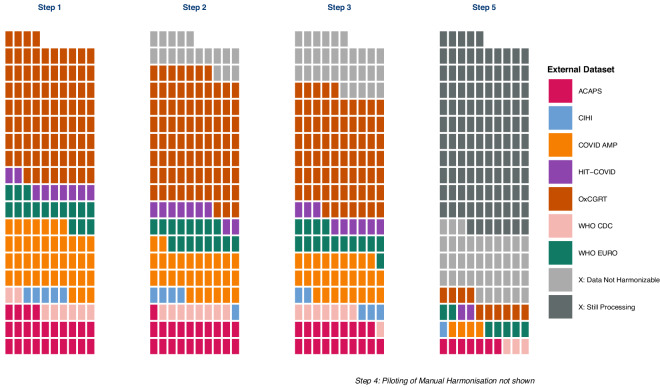
PHSM Data Harmonization Process. This figure visualizes the volume of data processed across the different steps of our harmonization process for a given PHSM dataset. During Step 1, the taxonomy for each dataset is mapped into the CoronaNet taxonomy and represents the absolute amount of data that is possible to harmonize. During Step 2, basic cleaning and subsetting of each dataset is performed in order to remove observations that are clearly not mappable into the CoronaNet taxonomy. During Step 3, an algorithm is employed to remove as many duplicate observations as possible. Step 4, not pictured, refers to our pilot harmonization efforts for select countries and datasets. Step 5 refers to our ongoing efforts to manually harmonize the remaining data. Please see the methodology section for more details.

The challenges posed by harmonizing multiple complex taxonomies of dirty data based on inconsistently preserved original sources led us to the conclusion that ultimately, only manual harmonization would allow us to harmonize data from different PHSM trackers in a way that would ensure high data quality and validity. Given the sheer number of policies in the external dataset however, to the extent possible, we sought to support these manual efforts with automated tools to harmonize data across 8 different datasets into the taxonomy for capturing COVID-19 PHSM developed by the CoronaNet Research Project. To that end, we followed the methodology laid out in Fig. 1. That is, after we evaluated the set of COVID-19 PHSM data to harmonize, we made taxonomy maps between the different external data and the CoronaNet taxonomy (Step 1), performed basic data cleaning as well as data trimming of policies from the external dataset irrelevant to the CoronaNet taxonomy (Step 2), and automatically deduplicated a portion of the external data (Step 3). After having piloted manual harmonization for a sample of the data (Step 4), we are currently manually harmonizing the remaining external data into the CoronaNet dataset (Step 5).

Table 6 provides a numerical breakdown of how different data have been processed along these steps and an overview of our harmonization efforts to date. It shows that after preprocessing and standardizing the raw data using automated methods (Step 1 through Step 3), there remain 150,052 policies from the 7 datasets external to CoronaNet to harmonize manually (Step 5). While Step 5 is still ongoing, more than 50% of the external policies have been assessed for whether they overlap with the CoronaNet data or not and around 36% has been assessed for harmonization into the CoronaNet dataset. Policies recoded into the CoronaNet dataset originally found from these external datasets can be identified in our publicly available dataset (see https://www.coronanet-project.org) using the ‘collab’ and ‘collab_id’ fields which refer to the external dataset source and original unique id respectively. For a fuller accounting of our data harmonization methodology, please see the methodology section of this article.

**Table 6:** State of External Data at different steps of the data harmonization process.

Dataset	Step 1	Step 2	Step 3	Step 5	
	Raw	Consistency	Automated	% Overlap	% Harmonization
	Data	Subset	Deduplication	Assessment	Assessment
	(#)	(#)	(#)	Completed	Completed
All Data	180842	162991	150052	53.65	36.46
ACAPS	23926	20842	18699	73.61	49.05
WHO CDC	7985	7405	7096	71.58	48.08
CIHI	4417	4235	4210	16.58	7.05
COVID AMP	39332	27703	26473	27.77	17.89
WHO EURO	15258	15071	14220	86.76	73.87
HIT-COVID	8917	8606	8142	48.54	30.51
OxCGRT	81007	79129	71212	50.69	28.76

### Assessing the Value of Harmonizing COVID-19 PHSM Data

Given the apparent intricacy of harmonizing data, to say nothing of harmonizing complex, unclean and incomplete data, it is easy to miss the forest for the trees. As such, in this section, we take a step back in order to provide an assessment of the overall value of harmonizing COVID-19 PHSM data. To do so, in the following we draw from guidance we developed in a separate commentary^12^ to explore the benefits, costs, limitations, necessary resources and alternatives to data harmonization of PHSM data.

#### What can be gained from data harmonization?

To our knowledge, no individual effort to document PHSM has been able to do so for all countries. Indeed, though at the time of writing, there are 145k+ observations unique to the CoronaNet dataset (of the 180k+ observations available in total, which includes harmonized data), we identified 150,052 observations for the 7 datasets external to CoronaNet combined for data available through September 2021. According to our efforts so far, around 83% of external data do not overlap with the CoronaNet dataset, and of these around 45% can be recoded, suggesting there are potentially 55k additional observations to recode.

**Fig. 2:**
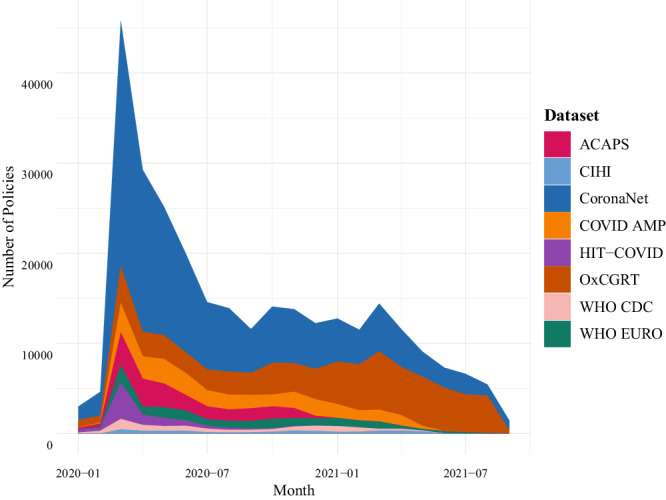
Number of policies per date recorded by 8 different COVID-19 PHSM tracking efforts.

Data harmonization would thus lead to a dataset that is more complete and consistently coded across time and space then is currently available. Indeed, Fig. 2 shows that while most datasets have fair coverage of PHSM until the summer of 2020, with data from CoronaNet being especially rich, data after this time is limited especially for trackers that stopped data collection (e.g. HIT-COVID, ACAPS). OxCGRT in comparison to other datasets, has been able to document more policies for later months.

**Fig. 3:**
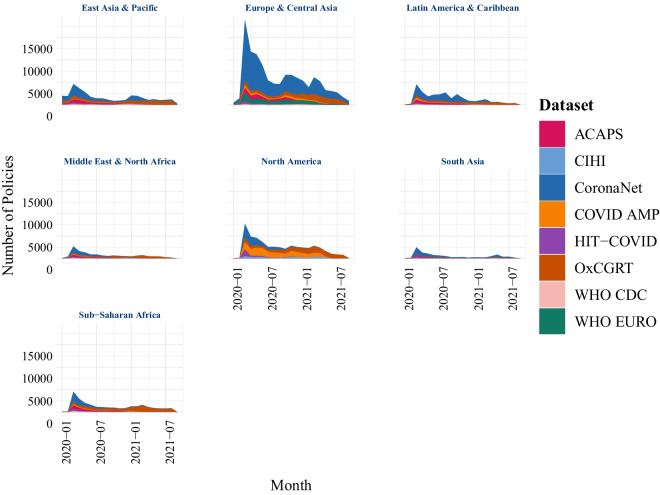
Number of policies per date, grouped by region, recorded by 8 different COVID-19 PHSM tracking efforts.

Meanwhile Fig. 3 illustrates differences in the number of policies captured across continents. Clearly, all trackers have asymmetrically focused on countries in Europe and North America.

While data harmonization cannot compensate for this relative unevenness in data coverage, it can significantly improve coverage of non-European and non-North American countries on an absolute level.

**Fig. 4:**
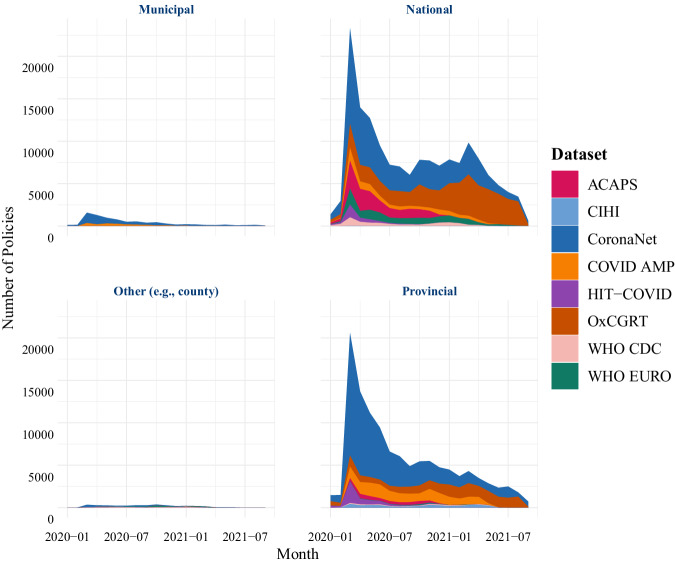
Number of policies per date, grouped by the initiating level of government, recorded by 8 different COVID-19 PHSM tracking efforts.

Moreover, as Fig. 4 shows, most external datasets either focus on gathering national-level data for countries around the world or subnational data for a more limited number of countries, but rarely both.

As such, data harmonization efforts will substantially improve the availability of PHSM data initiated at the national level and to some degree, the provincial level as well.

Overall, data harmonization greatly advances the completeness of PHSM data on a number of dimensions, including time, space, and administrative levels. Moreover, our data harmonization methodology also allows each policy in the external dataset to be evaluated independently, which can improve the quality of the PHSM data overall. This is all the more valuable given that while PHSM data has generally been made publicly accessible in close to real time because of the emergency nature of the pandemic, research groups have not been able to guarantee data cleanliness (see the ‘Challenges of Data Harmonization’ section). Progress on these dimensions greatly improve the research community’s ability to conduct analyses on the COVID-19 pandemic which can yield results with both greater external validity and generalizability (in e.g. cross national analyses) as well as analyses that can yield results with greater internal validity and with fewer potential confounders (in e.g. subnational analyses).

#### What can be lost from data harmonization?

The main loss when harmonizing PHSM data into the CoronaNet taxonomy is with regards to measures that CoronaNet does not capture and for which, the benefit of its relative fine-grained taxonomy are moot. The most prominent of these measures are the economic ones, such as business subsidies or rental support. For measures for which there is conceptual overlap between the CoronaNet taxonomy and other taxonomies, the fact that the data were harmonized to the CoronaNet taxonomy, which by far has the most detailed taxonomy of the 8 datasets, minimizes the extent to which information was lost from the harmonization process.

Meanwhile, the benefits of data harmonization aside, there can be real scientific value when different researchers approach similar research topics with different research designs^19^. In support of this, we further make taxonomy maps between the CoronaNet taxonomy and the taxonomy of each respective dataset publicly available through our Supplementary Information, Section 2. These maps can not only help users better understand how to use different datasets, but can also provide robustness checks of COVID-19 related research and bolster the transparency and replicability of our data harmonization efforts.

#### What are the limits of data harmonization?

While we believe that our efforts to harmonize data across 8 different datasets will provide the most complete picture possible of COVID-19 PHSM, they will still fall short of a dataset that will reflect all COVID-19 PHSM ever implemented. Though it is inherently impossible to assess how much data will still be missing after data harmonization is finalized — a complete dataset needs exist to make this assessment and it does not — we offer some insights as to where and why data may be incomplete. Specifically, our complete, harmonized dataset will still (i) lack information on subnational policy making for a number of countries as well as low state capacity governments and (ii) be unable to ensure complete data cleanliness.

**Fig. 5:**
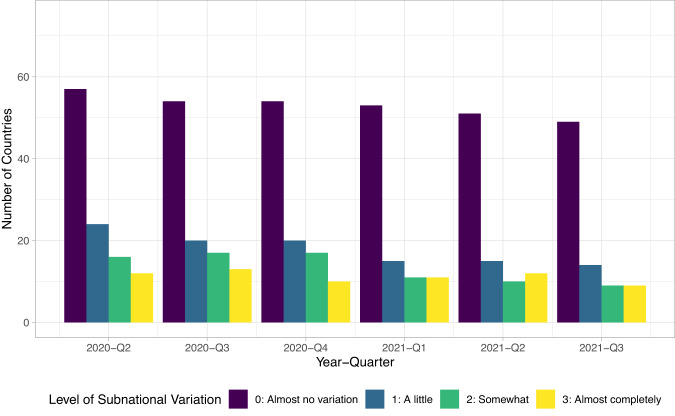
Extent of policies made at the subnational level by quarter, from CoronaNet Research Project internal assessment data.

**Fig. 6:**
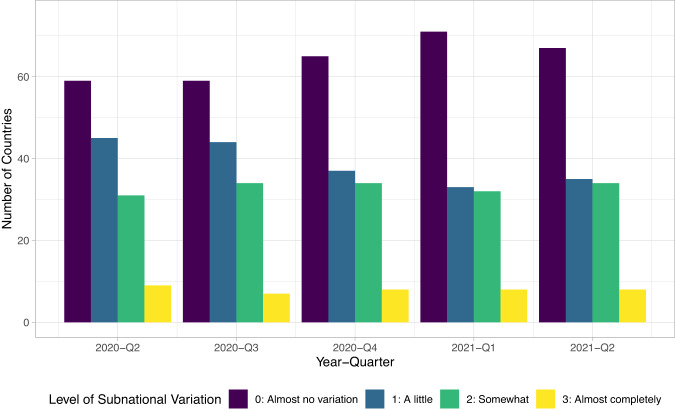
Extent of policies made at the subnational level by quarter, from the Varieties of Democracy Pandemic Backsliding Project (PanDem).

Our review of projects gathering COVID-19 policies suggests that most projects focus on national level policies, limiting what data harmonization can achieve. Table S1 in the Supplementary Information shows the coverage of data on subnational policy making for all datasets that we know to be in existence, using data available at the time of writing. Most datasets aside from CoronaNet do not collect subnational data and to the extent that they do, they overwhelmingly focus on the United States. Meanwhile, though the CoronaNet data does capture subnational data for some countries, given the volume of policies generated and limited resources, we are only able to capture this data for reduced time periods. However, available evidence suggests that subnational policy-making has taken place in many other countries beyond the ones listed in Table S1 in the Supplementary Information. Data from both the Varieties of Democracy Pandemic Backsliding Project (PanDem)^20^ as well as CoronaNet’s internal surveys suggest that there was subnational policy making in anywhere from 30 to 90 countries at any given point in time, as visualized in Figs. 5 and 6. Note that the CoronaNet internal surveys followed the same coding scheme as PanDem’s [subvar] variable; at the time of writing, CoronaNet’s internal assessment covers 115 countries for 6 quarters while PanDem’s data covers 144 countries for 5 quarters, with 91 countries covered in common across both.

Meanwhile, we also identify how issues of low state capacity can make it difficult to document COVID-19 policies at all. Some problems that CoronaNet researchers have reported include:

- No announcement of policies in any official government sources: In the absence of any official government sources about a policy, research assistants must rely on media reports which can often have conflicting information about the nature or timing of a given policy. It is also not uncommon for governments to announce policies on social media without providing further information in the form of official government sources.
- Policies being communicated in mediums other than the Internet: In places with low internet connectivity, governments have been known to make policy announcements in non-digital forms used most prevalently by the local population e.g. radio, local news bulletins, town criers.
- NGOs and/or IOs implementing policies that are normally under the purview of governments: When governments lack the capacity to respond the COVID-19 pandemic, NGOs or IOs have been known to step in. While it is possible to capture these policies, policy trackers to date have largely focused on documenting government initiated policies.

In short, large scale data collection efforts of PHSM data have been predicated on: (i) the capacity to capture PHSM polices made at all different administrative levels (ii) the availability, access and durability of web-based documentation on PHSM policies and (iii) the assumption that governments are the primary policy responders to the COVID-19 pandemic. However, these conditions are not always present in low state capacity states. While the enormous undertaking described here will greatly advance our collective knowledge of COVID-10 PHSM policies, much more funding and support is needed to document all PHSM.

Finally, as we elaborate more fully in the ‘Challenges of Data Harmonization’ section, PHSM data is unusually challenging to harmonize because the emergency nature of the pandemic gave rise to multiple complex taxonomies and corresponding datasets that have had varying levels of quality, completeness, and underlying source material.

While we employ some automated processes to harmonize taxonomies and deduplicate data, our methodology is overwhelming reliant on the analog process of recoding external data based on the original sources found in the external data rather than relying directly on the observations available in the external data itself. In doing so, we can ensure that whatever errors might have been made in the automated taxonomy harmonization processes, which itself was adjusted to account for systemic errors in the external data (see the ‘Step 1. Making Automated Taxonomy Maps’ section below), can be rectified manually later. Meanwhile we have also additionally vigorously tested our automated deduplication strategies to ensure that we are biased towards keeping duplicates to be removed later manually rather than mistakenly removing observations that are not duplicates (see the ‘Step 3. Automated Deduplication’ section below). However, despite out best efforts, we can nevertheless not guarantee that the likely 55k+ observations that we will eventually recode from the external datasets into the CoronaNet taxonomy will be completely free of error.

#### What cooperative resources are available for harmonizing data?

External data partners were either co-hosts or participants in the two conferences hosted by CoronaNet: the PHSM Data Coverage Conference (February and March 2021) and the PHSM Research Outcomes Conference (September 21, 2021). During both conferences though especially the first, trackers discussed common challenges and solutions to their data collection efforts, especially with regards to taxonomy and organization. Both the planning of the conferences and conferences themselves helped increase mutual understanding and collegiality among trackers^1^. For more information, please see https://covid19-conference.org or the shared statement written by conference participants outlining a framework for cooperation and collaboration (PHSM 2021).

Meanwhile, bilateral exchanges also played an important role in identifying and overcoming specific challenges with regards to mapping and harmonizing data for a given dataset. For instance, our ability to harmonize the CIHI dataset, was contingent on close cooperation with the CIHI team. Aside from explicit coordination on COVID-19 vaccines taxonomy, three volunteer researchers for CoronaNet were contracted to work on the CIHI database. This shared expertise greatly facilitated our ability to build a taxonomy map between CIHI and CoronaNet and to pilot our harmonization efforts.

Similarly, researchers from both CoronaNet and HIT-COVID were involved in building the HIT-COVID taxonomy map, which greatly facilitated the mapping process. They were also involved in piloting the data harmonization process, which also increased the speed at which it could be done. The fact that HIT-COVID and CoronaNet built their taxonomies for COVID-19 vaccine policies with mutual feedback from the other also facilitated the mapping of this particular policy type.

Meanwhile, ACAPS, COVID AMP, and OxCGRT generously made themselves available for clarifying confusion or misunderstandings about their respective taxonomies which helped make the mappings more accurate. However, despite repeat inquires to the WHO PHSM dataset to initiate such cooperation, we found them to be unresponsive which made the taxonomy mapping exercise with the WHO PHSM dataset comparatively difficult. Overall, we found that greater communication and cooperation between leaders of different datasets was an important intangible in facilitating the data harmonization process.

#### What are alternatives to data harmonization?

While in this paper we concentrate on presenting our rationale and methodology for qualitatively harmonizing PHSM data, in Kubinec et al. (2021) we introduce a Bayesian item response model to create policy intensity scores of 6 different policy areas (general social distancing, business restrictions, school restrictions, mask usage, health monitoring and health resources) which combines data from both CoronaNet and OxCGRT^21^. As this latter paper shows, researchers should be cognizant that while statistical harmonization can be an effective form of data harmonization, the resulting indices or measures may sometimes need to be interpreted or used differently than the underlying raw data. For example, our policy intensity scores for mask wearing can be interpreted as the amount of time, resources and effort that a given policy-maker has devoted to the issue of mask restrictions in a given country compared to that of other countries. This is different from what the underlying raw data measures: whether a given mask restriction is in place or not. Researchers choosing to engage in statistical harmonization should thus provide a thorough accounting of the underlying concept that they seek to measure and a corresponding justification of why their statistical method provides a good operationalization of it.

## Discussion

This article presents our efforts to harmonize COVID-19 PHSM data for the 8 largest existing datasets into a coherent, unified one, based on the taxonomy developed by the CoronaNet Research Project. To do so, we provide a thorough accounting of the various challenges we faced in harmonizing such data as well as the methodology we used to address these challenges. Along the way, we open a window into understanding the strengths and weaknesses of different COVID-19 PHSM datasets and create a new path for future researchers interested in harmonizing data more generally to follow.

We also show that there are substantial gains to harmonizing PHSM data across 8 different datasets, particularly in terms of the time, spatial and administrative coverage of PHSM data. While some conceptual diversity is always lost when harmonizing data, we argue that by harmonizing PHSM data to the CoronaNet taxonomy, this issue is minimized due to the CoronaNet taxonomy’s comparative richness. Data harmonization of these 8 datasets will still fall short of a complete PHSM dataset, especially for countries for which there is a great deal of subnational policy making or low state capacity but this effort nevertheless will provide the fullest picture yet of COVID-19 government policy making. Moreover, it substantially improves upon the existing WHO PHSM effort to harmonize data both in terms of scale and quality (see Supplementary Information, Section 3). More resources would allow us to complete data harmonization more quickly, which given the ongoing nature of the COVID-19 pandemic, would be welcome. However, even if data harmonization is completed only after the pandemic is overcome, it will still present a tremendous historical resource for generations of researchers.

Our experience in data harmonization has underscored for us that the production of data may be understood not only as a mere reflection of reality, but a framing or even creation of reality. That is, by producing certain measures and not others, data can frame certain aspects of the world as more or less deserving of attention. Meanwhile creating a measure in the first place can bring forth concepts that previously did not exist in the public consciousness^22^. Harmonizing data cannot escape these dynamics and in fact invites greater scrutiny of them as it adds another layer of negotiation and complexity in terms of determining what is worthy of being measured and how to measure it. Undergirding all of this are social processes that produce data in the first place and which can have important influence on what data ultimately is or is not harmonized^23^. Though in a number of fields, researchers have developed novel platforms that aim to help facilitate data harmonization^24,25^, ultimately effective data harmonization requires researchers to identify clear goals for their harmonization process, a high level of attention to detail in designing a rigorous plan to carry out, and a robust working culture to ultimately successfully implement it. We hope our experience with PHSM data harmonization can provide a roadmap for researchers embarking on similar journeys for their own research.

## Methodology

### COVID-19 PHSM Harmonization Methodology

In this section, we provide greater detail for the 5 step methodology we employed to semi-manually harmonize data from 7 PHSM datasets into the CoronaNet taxonomy for policies implemented by governments before September 21, 2021. We start with outlining each of these different steps before expanding on each step in separate subsections later on. A note to the reader: unless explicitly noted, any subsequent analysis or description of the external data refers to data recorded by September 21, 2021.

1. Step 1: Create taxonomy maps for each external dataset and CoronaNet, which we make publicly available (see Supplementary Information, Section 2). Based on these maps, we then mapped data available for each external dataset into the CoronaNet taxonomy
2. Step 2: Perform basic cleaning and subsetting of external data to only observations clearly relevant to existing CoronaNet data collection efforts.
3. Step 3: Remove a portion of duplicated policies using customized automated algorithms with respect to:
  Duplication within each respective external dataset
  Duplication across the different external datasets
4. Step 4: Pilot our data harmonization efforts for a select few countries (over the summer of 2021)
5. Step 5: Release the resulting curated external data to our community of volunteer research assistants to:
  Manually assess the overlap between PHSM data found in CoronaNet with that found in the ACAPS, COVID AMP, CIHI, HIT-COVID, OxCGRT, the WHO EURO and WHO CDC datasets respectively and;
  Manually recode data found in the external datasets that were not already in the CoronaNet dataset into the CoronaNet taxonomy.

### Step 1. Making Automated Taxonomy Maps

Given the variety and complexity of approaches that different groups have taken to document PHSM policies, asking research assistants to not only become experts in one taxonomy but multiple taxonomies would have been unfeasible. Instead, we created maps between the CoronaNet taxonomy and other datasets so that all datasets could be understood in the CoronaNet taxonomy for the following principal fields:

- Policy timing
  - The start date of the policy
  - When available, the end date of policy
- Policy initiator
  - The country from which a policy is initiated from
  - When available, the ISO-2 level region from which a policy is initiated from
- Policy Type
  - Broad policy type
  - When possible, the policy sub type
- Sources/URLs
  - URL links
  - When available, links of original pdfs
- Textual description

When possible, other fields, such as the geographic and demographic targets, are also matched. As outlined in the ‘Challenges of Data Harmonization’ section, because of conceptual and organizational differences across different taxonomies, one to one mappings were not always possible especially with regards to the substance of COVID-19 policies. In such cases, one to two or one to three mappings were suggested. For the COVID AMP and WHO PHSM mappings (relevant for the WHO EURO and WHO CDC datasets), we also employed machine learning models to predict the most likely policy type an observation was likely to be in the CoronaNet taxonomy based on the textual description of the policy. Both because one to one mappings based on the taxonomies themselves were often not possible and because of issues with dirty data, in some cases, the mappings were often adjusted to so that they were based not only on the formal taxonomy but also on when certain key words were used in the dataset. For example, though policies originally coded in the WHO taxonomy of ‘Social and physical distancing measures (Category) - Domestic Travel (Sub-Category) - Closing internal land borders (Measure)’ might reasonably map onto CoronaNet’s ‘Internal Border Restriction’ policy type, when the word ‘quarantine’ appears in the text description of such policies, we reclassify them in the taxonomy map as a ‘Quarantine’ policy instead. As such, these taxonomy mappings are not always based strictly on how different policy types theoretically should map onto each other, but attempt to account for mistakes and miscodings in the external data to create the best mapping possible between the existing data and the CoronaNet dataset. In this first automated step, our aim was to ensure that *most* mappings were correctly mapped but did not take pains to make sure that *every* mapping was correctly mapped, because, as we explain later on, each observation was ultimately assessed and evaluated for harmonization by human coders who are better equipped to make these more fine-grained and nuanced judgements.

As part of this mapping exercise, in order to keep track of the original dataset that each observation came from, we also ensured that each record was associated with its own unique identifier (unique_id). In some cases, reformatting the data also impacted how the unique_id assigned by the original dataset was formatted, though we ensured that our transformation method nevertheless still allows others to trace a given observation back to the original dataset. For example, in HIT-COVID, border restrictions for people leaving or entering a country are coded in separate observations. However, in CoronaNet, if a policy for restricting both entry and exit to or from the same countr(ies) on the same date, they are coded as one observation. In this case, the HIT-COVID data is collapsed to fit into one observation and the unique identifier is also collapsed such that two or more of the original unique identifiers are collapsed into one when they are mapped to the CoronaNet taxonomy. In the case of OxCGRT, no unique identifiers are provided in the original dataset and in this case we create them using a combination of the policy indicator, date, country and where applicable, province.

Please see the Supplementary Information, Section 2, for more information about how to access the specific taxonomy mappings we created between CoronaNet and other datasets.

### Step 2. Basic cleaning and subsetting of external data

With the help of the taxonomy maps, we were able to roughly transform the external datasets into the CoronaNet taxonomy. Before moving forward with manual data harmonization, we first implemented some basic cleaning and subsetting of the data. Because, as discussed in the ‘Challenges of harmonizing different taxonomies’ subsection above, most datasets do not use a consistent reference for identifying policies originating from the ISO-2 provincial level, we created code to clean these text strings up as much as possible. Given the sheer number of observations that needed such cleaning, we could not ensure full standardization for these text strings. However, we took pains to ensure that the 430+ provinces for which CoronaNet systematically seeks to collect subnational data for were consistently documented in the external data. Specifically, these are subnational provinces for the following countries: Brazil, China, Canada, France, Germany, India, Italy, Japan, Nigeria, Russia, Spain, Switzerland, and the United States.

Next we subset the external data to exclude regions that CoronaNet is currently not collecting data for. In particular, we excluded observations from the COVID AMP dataset documented at the county or tribal level in the United States as well as observations for Greenland, the United States Virgin Islands and Guam from our harmonization efforts. In addition, we also subset the external dataset to exclude policy types that CoronaNet is currently not collecting data for, in particular economic or financial measures taken in response to the pandemic.

### Step 3. Automated Deduplication

After making taxonomy maps for each external dataset to the CoronaNet taxonomy and conducting some basic cleaning of the data, we also took steps to deduplicate the data using automatic methods to the extent possible. Deduplication was assessed along three criteria: i) duplicates within each external dataset ii) duplicates across the external datasets and iii) duplicates between the CoronaNet and external datasets. We outline the steps we took to assess the level of duplication along each of these criteria and when possible, to remove duplicates accordingly. All in all, we took a conservative approach in our automated deduplication efforts insofar as we rather left many potential duplicates in the dataset rather than removed too many policies which may have not been duplicates.

### Step 3a. Deduplication within External Datasets

Given the sheer amount of data collected and coordination needed to collect such data, it is not surprising that there is some duplication within datasets. Duplicates can occur for a number of reasons including (i) structural differences between taxonomies (ii) the lack of one to one matching between taxonomies (e.g. a policy that may be coded as several policies in one taxonomy may only be coded as one policy in the CoronaNet taxonomy) (iii) coder error.

We first needed to deal specifically with duplication that occurs as a result OxCGRT’s method of collecting data to fit a panel data. In particular, OxCGRT coders are generally instructed to provide an assessment of whether a policy was in place or not for each given day that they are either recording the policy or for which they have evidence for a policy being in place or not. For instance, if a coder finds that the same policy has been in place over several weeks, the same textual description may be copied and pasted into the notes section of the dataset for each day that the coder happened to review the status of policy-making for that indicator, even if the ordinal indicator itself does not change. When initially extracting and reshaping the OxCGRT data into an event dataset format, each textual description is initially retained, even though it may not contain new information. To deal with this, we built a custom function to identify policies that repeated the exact same description, keeping the ‘latest’ instance of the policy description and removing earlier ones (see the OxCGRT-CoronaNet taxonomy map available through the Supplementary Information, Section 2, for more detail).

We also needed to implement a custom procedure to deal with a related practice of documenting ‘no change’ in a policy indicator which was unique to OxCGRT’s methodology for documenting policies. Specifically, when an OxCGRT coder does not identify any change in a policy indicator, it is customary for the coder to note something to the effect of ‘no change’ in the textual description for that particular day. This information can be extremely valuable if one desires to know the status of a given indicator in the ‘present’ as it allows researchers to distinguish whether there was truly no change in government policy makers or whether there was simply no one actively documenting government policy making for a given region and indicator. As the present becomes the past however, this information becomes less useful. For instance, while the value of knowing that there was ‘no change’ in a given indicator ‘today’ is quite high, knowing that there was ‘no change’ for a given indicator in e.g. March 2020 is not very informative especially if there was subsequently a flurry of policy making activity for that indicator. Given that we initially retained each textual description from the OxCGRT data when transforming it from a panel to event dataset format, our initial efforts created an OxCGRT event dataset format filled with observations that documented variations of the sentiment ‘no change.’ Because the CoronaNet taxonomy does not document when there are no policy changes, to the extent possible then, we sought to remove such observations from the OxCGRT dataset. The difficulty in doing so was compounded by the fact that (i) there appears to be no standard language that OxCGRT coders follow in communicating that a policy had no change (ii) not infrequently, a textual description will start by noting that there has been no change to a policy, but will then subsequently provide a long and detailed description of the policy. In these cases, it is unclear whether there actually was no change to a policy and the coder is simply noting what the policy was or if there was no change to the policy that could be captured by the OxCGRT taxonomy, but there were actually some changes made by the government and the coder is documenting them qualitatively in the text. To deal with the former issue, we looked through hundreds of OxCGRT policies to try to identify as many phrases that conveyed the sentiment ‘no change’ as possible. To deal with the latter issue, we did not remove observations over a certain character limit even when they noted that there was ‘no change’ in case there actually was a substantive change that could be captured in the CoronaNet taxonomy. These choices were consistent with our general conservative approach towards automated deduplication.

Following this specialized deduplication for the OxCGRT dataset, we then sought to identify duplicates within each dataset more generally. We experimented with identifying policies that had identical values for a variety of different policies and ultimately found the following set of variables as being able to accurately identify a large number of duplicates:

- description: records the textual description used to describe each observation. Note, for the purposes of deduplication, the descriptions were stripped of punctuation and special characters and transformed to all lower cased letters in order to decrease the likelihood that stray superfluous symbols would prevent the identification of duplicates. During the manual harmonization stage, the original descriptions are used.
- country: records the country that a policy originates from, where the list of countries are standardized.
- province: records the province that a policy originates from, where the list of policies are semi-standardized (see the ‘Step 2. Basic cleaning and subestting of external data’ subsection for more information).
- link: records the URL link used as the raw source of information for a given policy.

Theoretically, we believed that the likelihood of identifying true duplicates with the above variable fields are quite high given that all descriptions are all written in free form and that URL links can act as fairly robust unique identifiers. With this set of variables, we identified 6955 policies that were duplicated (note that we excluded from this procedure, policies that had the textual description ‘Extension’ or ‘extend’ in their descriptions. As part of our investigation, we found that it was common for coders to copy and paste the same description with this word every time a policy was extended in time and as such we would have inaccurately removed many policies had we not excluded such observations from our deduplication efforts). To assess our efforts, we sampled 100 groups of policies that were found to be duplicates, (which was equivalent to 393 total observations), and through manual investigation, found that 99 of these groupings were indeed duplicates, for an accuracy of 99%. We further manually checked groups of policies that were identified as having particularly high number of duplicates (7 or more, the maximum being 19) and found that our criterion accurately identified these groups of policies as having duplicates. Because this automated deduplication method proved to be quite accurate, we subsequently used this criterion to remove likely duplicates within each dataset. We show the distribution of policies we found to be duplicates according to this criterion in Table 7.

**Table 7:** Assessment of duplicates within datasets.

ACAPS	WHO CDC	CIHI	COVID AMP^1^	WHO EURO^2^	HIT-COVID	OxCGRT^3^
246	45	1	437	304	373	5549

This table shows the number of duplicate observations found within a given dataset (e.g. we identified 246 duplicate observations within the ACAPS dataset).

^1^We found that many duplicates for COVID AMP were due to the fact in some cases, when the same policy was used to target different cities, the same description, country, province and link were used. Because the CoronaNet taxonomy documents different target cities within one observation instead of multiple observations, when deduplicating this data, the code was adjusted such that the information on the target cities in the COVID AMP data was aggregated and preserved in the observation that was kept while the other observations were discarded.

^2^We found that many duplicates for the WHO EURO data was due to the fact that policy sub types were recorded as separate observations while using the same description, country, province and link. Since initial taxonomy mapping exercise was often only precise to the policy type, not the policy sub type, it was fine to discard these observations as duplicates given that the core information would still be retained and manually recoded more precisely in terms of policy sub types by human coders later on.

^3^Duplicates in the OxCGRT data were found for a number of reasons including (i) often, coders would use the same description and links across separate OxCGRT indicators for a given country and start date. Because these policy types were already mapped as a ‘one to many’ policy type mapping, it was fine to discard these policies as duplicates. (ii) A fair number of policies are ‘no change’ policies that were not previously caught in the custom procedure described to capture such policies (iii) A fair number of descriptions only had a link in the description.

As can be seen, we identified a particularly high number of duplicates within the OxCGRT dataset. This is consistent with our knowledge that duplication is a particular problem with OxCGRT data because of their methodology for data collection as well as what we know to be a conservative approach in our custom method of deduplicating OxCGRT data.

### Step 3b. Deduplication across External Datasets

The data was also evaluated for duplicates across datasets. Data duplication across datasets happens because different policy trackers have only coordinated their work in collecting PHSM data to a limited extent. As such, the same policy may be independently documented by coders in different datasets. While this is desirable from the point of view of data validation, it is a hindrance from the point of view of data harmonization.

As a first step in deduplicating data across datasets, we were able to remove a number of observations that were by definition duplicates. Specifically, since the OxCGRT subnational data for Canada is based in large part on the data collected by CIHI, we removed OxCGRT data for Canada from our dataset and instead chose to prioritize the more fine-grained version of the data documented by the CIHI dataset. Note that the full WHO PHSM dataset actually includes data from ACAPS, HIT-COVID and OxCGRT. These observations were removed from the dataset as well following similar a logic. That is, it seemed likely that a direct translation from e.g. the ACAPS to CoronaNet taxonomy would lead to fewer errors than using the version of the data that first translates ACAPS to the WHO PHSM taxonomy and then to the CoronaNet taxonomy. Second, it further allows us to maintain evaluate the full ACAPS, HIT-COVID and OxCGRT datasets; whereas in the WHO PHSM dataset, the ACAPS, HIT-COVID and OxCGRT data has already been deduplicated according to the WHO PHSM methodology.

Following this, we then experimented with identifying duplicates across datasets more generally. In addition to exploring which set of variables most reliably identified groups of true duplicates (as we did for identifying duplicates within datasets), when duplicating across datasets, we further had to decide from which dataset observations should be retained when duplicates were found. With regards the former, we found that identifying duplicates based on the following variables to yield the most accurate results:

- type: records the broad policy area of a given COVID-19 policy. E.g. a policy related to schools will be coded as ‘Closure and Regulation of Schools’ type.
- type_sub_cat: The specific policy area of a given COVID-19 policy. This is hierarchically determined such that only certain type and type_sub_cat combinations can go together. E.g. a policy related to primary schools will have a type_sub_cat of ‘Primary Schools’ and will by definition have a policy type of ‘Closure and Regulation of Schools.’
- country: records the country that a policy originates from, where the list of countries are standardized.
- province: records the province that a policy originates from, where the list of policies are semi-standardized (see the ‘Step 2. Basic cleaning and subsetting of external data’ subsection for more information).
- target_who_what: if applicable, records the citizenship (citizen or non-citizen) or travel status (traveller or resident) which a given policy is targeted toward.
- date_start: records the start date of a given policy.
  Note, we considered other variables but found that they were not adequate because they were not broadly collected across different external datasets. E.g. enforcer is only collected by CIHI; target_country, target_province is only collected by COVID AMP; target_direction and institution_status are only collected by HIT-COVID; type_mass_gathering is only collected by WHO EURO and WHO CDC; date_announced is only collected by COVID AMP and CIHI.
  Meanwhile, with regards to the issue of what observations we should ultimately retain when duplicates were identified, we developed a protocol for prioritizing datasets based on both our qualitative experience working and transforming each dataset during the taxonomy mapping exercise in Step 1 as well as the quantitative assessment of the data quality of each dataset which we outlined in the ‘Challenges of Data Harmonization’ section. When there was only one duplicate identified for a given observation, we chose to retain information from the dataset that had the most number of characters in its textual description of that observation. When more than one duplicate was identified per grouping however, we developed the following protocol for prioritizing which observation to retain:
- Priority 1: For Canadian data, CIHI is prioritized first because this dataset specializes in collecting Canadian data.
- Priority 2: COVID AMP is prioritized second for all data except for Canadian data based on both our qualitative and quantitative assessment of COVID AMP data quality. Based on our experience creating the taxonomy map between COVID AMP and CoronaNet, we found that COVID AMP’s taxonomy was very similar to the CoronaNet taxonomy, mitigating the challenge of taxonomy mapping and potential attendant errors. In terms of our quantitative assessment of COVID AMP data quality, we found it to be relatively high quality insofar as there are very few missing links and relatively high quality textual descriptions. Note however, that the version of the COVID AMP data we harmonized available in 2021 only collects data for 64 sovereign countries (while 95 are available in its dataset, these include policies for United States Native American tribes).
- Priority 3: WHO CDC and WHO EURO is prioritized third for all data except for Canadian data. These data were prioritized together because they have already been mutually assessed for deduplication within the WHO PHSM dataset. In terms of data quality, the WHO CDC data appears to have higher quality descriptions compared to OxCGRT, ACAPS and HIT-COVID based on the average length of the description, the number of descriptions with less than 50 characters, while the WHO EURO data appears to have higher quality descriptions than ACAPS and HIT-COVID based on the average description length and higher quality descriptions than ACAPS, HIT-COVID and OxCGRT based on the number of descriptions with less than 50 characters. Meanwhile, both datasets furthermore have fewer missing end dates than ACAPS, HIT-COVID and OxCGRT.
- Priority 4: OxCGRT is prioritized fourth for all data except for Canadian data because OxCGRT has some information on end dates and based on our qualitative assessment, has more informative descriptions of policies than HIT-COVID and ACAPS. This is supported quantitatively as well given that OxCGRT descriptions are on average longer and have less missingness than HIT-COVID and ACAPS descriptions.
- Priority 5: HIT-COVID is prioritized fifth for all data except for Canadian data because compared to the ACAPS taxonomy, the HIT-COVID taxonomy is relatively similar to the CoronaNet taxonomy and it is relatively rich in subnational data. It was prioritized after the other datasets in part because it has no information on end dates.
- Priority 6: ACAPS is prioritized sixth for all data except for Canadian data this is because the compared to the other datasets, its textual descriptions are of poorer quality and because it has no information on end dates.

Using the above methodology, we identified 5989 duplicate observations. The distribution of policies identified as duplicates is shown in Table 8. Here we can see that observations from OxCGRT and ACAPS were discarded most often given these criteria. We then sampled 100 groups of observations identified to be duplicates, for a total of 425 observations, using this algorithm and found that 74.5% to be true duplicates, meaning that likely, around 1500 observations were discarded as being duplicates in this process that likely were unique observations. Given that we identified around 180k observations to harmonize to begin with and that most policies discarded were from datasets that we had previously found to have a higher likelihood of duplication (OxCGRT) or to be comparatively of lower quality (ACAPS), we made the judgement call that it was acceptable to discard this small percentage of observations without threatening the rigor of the data harmonization exercise writ large. Moreover, discarding these policies for consideration for manual harmonization at this point does not preclude doing so at a later state should resources allow for reassessing the value of harmonizing these policies.

**Table 8:** Assessment of duplicates across datasets.

ACAPS	WHO CDC	CIHI	COVID AMP	WHO EURO	HIT-COVID	OxCGRT
1909	273	22	753	519	92	2421

This table shows the number of duplicate observations found across a given dataset (e.g. we identified 1909 observations in the ACAPS dataset which was found to duplicate information found in at least one non-ACAPS dataset).

### Step 3c. Deduplication between CoronaNet and External Datasets

Lastly, we also evaluated the extent to which there were duplicates between the CoronaNet dataset and the external datasets. Such duplication can occur for the same reason that there is duplication across datasets: there has not been coordination between CoronaNet and these other datasets in terms of collecting policies and as such it is quite possible that there are duplicates across these datasets.

Like our attempts to identify duplicates both within and across the external datasets, we also experimented with different sets of variables that could accurately identify true duplicates across the CoronaNet and external datasets. However, ultimately we were not able to find a combination that yielded sufficiently high accuracy. Our best attempt used the following variables to identify true duplicates:

- country
- province
- date_start
- init_country_level
- link

Based on this criteria, we sampled 100 groups of policies found to be duplicates (equivalent to 764 observations) but found that only 14 were true duplicates, for an accuracy of 14%. Subsequent efforts with other sets of variables did not improve on this percentage. As such, we were unable to automate deduplication of the external dataset across the external and CoronaNet datasets and limited our automated deduplication efforts to deduplication within and across external datasets.

As a last step, we adjusted the dataset at this stage for the sample of policies that we manually inspected for duplication in Steps 3a and 3b. In other words, we recovered the policies that the algorithm falsely identified as being duplicates and added them back to the dataset to be evaluated for manual harmonization. In so doing, we additionally identified observations that would not be considered policies in CoronaNet from this sample (around 50) and removed them for consideration from manual harmonization.

### Step 4. Piloting of Manual harmonization Efforts

Steps 1 through 3 yielded an external dataset for which automated taxonomy mappings provided a rough first translation of the external data to the CoronaNet taxonomy and automated deduplication was able to remove the most obvious instances of duplicates within the external dataset.

As the challenges of harmonizing data from different, unclean data with inconsistently preserved raw sources revealed themselves, it became clear that the bulk of the work in data harmonization would need to be manual. While automated methods were able to reduce the size of the external dataset from around 180k to around 150k records, this still represents a tremendous number of policies to harmonize. To meet this challenge, the CoronaNet Research Project has been fortunate to be able to recruit hundreds of volunteers from around the world to help us complete this task.

Before rolling out these efforts to the entire project however, we first piloted data harmomization for a subset of each external dataset in order to i) validate the accuracy of the automated taxonomy mappings in Step 1 and ii) learn about potential difficulties and pitfalls as well as useful strategies to data harmonization so as to provide better guidance to future volunteers.

Table 9 describes the scope of our pilot harmonization efforts. The ‘assessment time frame’ refers to the actual time frame spent on piloting the data harmonization efforts (as opposed to the when the policies themselves were implemented). Part of the reason for these staggered time frames is that each taxonomy map itself took around 3-4 weeks to create; once a taxonomy map was created, it was immediately piloted for a given geographical scope. The choice to pilot certain countries and regions depended both on the availability of data for a given region for a given dataset and CoronaNet’s own prioritization of harmonizing European countries first given its partial funding from an EU Horizon 2020 grant. While relatively more assessments were done for taxonomies that were piloted earlier, fewer policies were assessed later on in part because i) taxonomy maps became better given the experience building the earlier ones and ii) assessment capabilities became higher given the experience of assessing earlier taxonomies. The order of mapping taxonomies from certain datasets as opposed to others was largely a function of how much capacity for cooperation the partner dataset was able to provide in building a given taxonomy map.

**Table 9:** Assessment Pilot harmonization Efforts.

Dataset	Assessment Time Frame	Geographical Scope	No. Policies Assessed
CCCSL	March to April 2021	Liechtenstein, United Kingdom	approx 600
HIT-COVID	May to August 2021	India subnational (Andhra Pradesh, Bihar, Chandigarh, Chhattisgarh, Dadra and Nagar Haveli, Goa, Gujarat, Hayana, Jammu and Kashmir, Madha Pradesh, Puducherry, Punjab, Tamil Nadu, Tripura, Uttar Pradesh), Slovenia, Luxembourg	687
CIHI	July to September 2021	Saskatchewan, New Brunswick, Alberta, Manitoba	469
WHO CDC and WHO EURO	June to September 2021	Slovenia, North Macedonia, Estonia	330
COVID AMP	August to September 2021	United States subnational (North Carolina, Maryland, Wyoming, Alaska, Georgia)	262
OxCGRT	September 2021	Luxembourg	68
ACAPS	September 2021	Bulgaria	40

As can be seen in Table 9, we initially sought to also include CCCSL in our pilot harmonization efforts. Unlike other taxonomy maps, the taxonomy map in this case was spearheaded by CCCSL partners. However, as part of the pilot assessment exercise, we found that both the CoronaNet and CCCSL were too complex to create high-accuracy maps. As previously discussed, given that CCCSL also had only around 11k observations, relatively few observations compared to other trackers with aspirations to track policies world-wide, inconsistently preserved sources, and unstandardized descriptions, we decided to depriortize harmonizing CCCSL data.

In piloting this data harmonization process more generally, research assistants reported that vague or incomplete descriptions and missing or dead links increased the difficulty of the work. It was not uncommon to encounter duplicate policies or external policies that needed to be broken down into smaller pieces in order to translate properly into the CoronaNet taxonomy. The pilot harmonization process also produced a pool of strategies and tips that future research assistants could draw on in their own efforts. Some strategies include (i) reading through the descriptions of all observations for a given country or region first in order to catch potential errors in the dataset (ii) using the Way Back Machine to recover dead links (iii) being aware that national level data from the OxCGRT dataset may include information about subnational policies because of the particulars of their methodology. Ultimately, these experiences helped us finalize the procedure we developed to manually harmonize the data, which we describe more in the following section.

### Step 5: Manual harmonization of data

After having piloted our manual data harmonization efforts for each external dataset separately, we then finalized our plans for manual harmonization of the full combined external dataset into two main steps. First, each observation is assessed for whether it is already documented within the CoronaNet dataset or not. This information is saved internally under the column name ‘overlap_assessment.’ Second, observations that are currently not in CoronaNet are recoded using the CoronaNet taxonomy and harmonized into the CoronaNet dataset. This information is saved internally under the column name ‘harmonize_assessment.’ We elaborate on each of these steps in the below.

In order to allow coders to manually assess the external data according to this criterion, we wrote the external data into Google Sheets, which we refer to here as the ‘Data Harmonization Sheets’ (referred to internally as ‘Data Integration Sheets’) We grouped each sheet by country or subnational region and added conditional formatting to help facilitate their assessments. A note here on language: at the beginning of our harmonization process, we inaccurately referred to our efforts as ‘data integration’ instead of ‘data harmonization.’ To reduce confusion for the reader, we use the term ‘harmonization’ instead of ‘integration’ in what follows. We report this discrepancy here for the sake of transparency.

By using Google Sheets, we were able to provide an editable, centralized place for numerous different people to assess the external data. In addition to the ‘overlap_assessment’ and ‘harmonize_assessment’ columns as well as columns to record which human coder made a given assessment, these sheets also provide information about the:

- Unique identifier for a given external observation (unique_id)
- Dataset that it belongs to (dataset)
- Textual description of the observation (description)
- Timing of the policy (date_start; date_end)
- Likely policy type. The type and type_sub_cat: provides the direct mapping while type_alt and type_alt_2 provides the machine learning prediction of the policy type, when available
- Demographic targets of a policy when available (target_who_what, target_who_gen)
- Geographic information about the policy initiator (country, province, city, init_other)
- Geographic target of the policy (target_country, target_province, target_city, target_other)
- Compliance of the policy (compliance)
- Types of travel the policy affected if applicable (travel_mechanism), and
- Raw source of the policy either in terms of the original URL (link) or a PDF of the source (pdf_link)

We summarize each of the steps below before then providing an example of how the Data Harmonization Sheets are used following this methodology. Though manual harmonization of the data is still ongoing, we close the section by providing an assessment of our progress to date and a discussion of tools and resources we have developed to support this process.

### Step 5a. Manual assessment of overlap between external and CoronaNet data

For each observation in the external dataset, a human coder evaluates whether this observation has previously captured in the CoronaNet dataset or not. This evaluation is stored in the column ‘overlap_assess’ in the Data Harmonization Sheets and can take on the values of ‘Yes’, ‘No’, or ‘NA.’ The meaning of each of these values is as follows:

- ‘*Yes*’: this means that the external observation had already been independently captured in the CoronaNet dataset. In this case, the research assistant copies and pastes the corresponding CoronaNet unique identifier, which is stored in its record_id variable, into the matched_record_id column in the Data Harmonization Sheet.
- ‘*No*’: this means that the external observation has not been previously captured in the CoronaNet dataset. In this case, the human coder should move onto the second step of manually harmonizing the data.
- ‘*NA*’: this means that no one has yet been able to make an assessment of whether a given observation is or is not already in the CoronaNet dataset.

### Step 5b. Manual harmonization of data

If a given observation is found to be in the external dataset but not in the CoronaNet dataset, the human coder moves onto to the second step of harmonizing external data into the CoronaNet taxonomy. To do so, they are instructed to treat the external observation just as they would any other potential source of information about a COVID-19 policy. In particular, they are asked to first go to the raw source of information using either the URL or PDF links (if available) provided for a given policy. Put differently, they are asked to recode the data based on the raw source of information provided in the Data Harmonization Sheets rather than from the textual description of the observation provided by the external data.

Once they have read through the raw information source, they can then either recode the information into the CoronaNet taxonomy using the normal procedure for documenting policies at CoronaNet (that is, they can recode this information into a Qualtrics survey customized for this purpose. See the methodology section in^6^ for more information) or they can provide another assessment of the external data. In the ‘harmonize_assess’ column, they can make one of the following 6 assessments:

- ‘Harmonized’; this means that the coder has recoded it into the CoronaNet taxonomy.
- ‘Harmonized with additional original research’: this means that the coder had to do some additional research before coding the observation into the CoronaNet taxonomy. This could be for any number of reasons. E.g. the information from the URL or PDF links in the external dataset may be unclear or require additional context/knowledge to code well.
- ‘Harmonized with additional work to find a new link’ means that the original link for the policy is dead but that the research assistant was able to find a new link that corroborates the information described in the ‘description’ column.
- ‘Harmonized with additional original research AND with additional work to find a new link: means the research assistant fulfilled both the criterion under: ‘Harmonized with additional original research’ and ‘Harmonized with additional work to find a new link.’ See above for more information.
- ‘Duplicated policy’: means that there were multiple external policies that were duplicates of each other. In this case, the research assistant is asked to only harmonize one of them and to mark the other ones as being duplicates.
- ‘Not a relevant policy’: means that after having taken a closer look at the link, the observation is not one that would be coded in the CoronaNet taxonomy.
- ‘Link dead, no other link found’: means that the original link for the policy as noted in the CoronaNet Data harmonization sheet is dead and the research assistant was unable to i) use the Way Back Machine to find the original data ii) find another link to corroborate this information. In this case, the research assistant is instructed to not recode this policy.

Table 10 provides a visual example of this data harmonization exercise for three policies in Hungary. The first policy was found to not have been in the CoronaNet dataset. As such, the coder marked the overlap_assessment as ‘No’ (unique_id: OXCGRT_Hungary_20210728_mask). After looking through the URL or PDF link (not shown in Table 10 but available for each observation in the harmonization sheet), the coder then subsequently assessed the policy as being an irrelevant policy to the CoronaNet dataset and thus ‘Not a relevant policy’ was chosen in the harmonize_assessment column.

**Table 10:** Example of Data Harmonization Sheets for Hungary.

overlap_assessment	matched_ record_ id	Notes	harmonize_ assessment	unique_ id	dataset	description
No	NA	NA	Not a relevant policy	OXCGRT_Hungary_20210728	OXCGRT	No policy changes. “It is no longer mandatory to wear a face mask, except in hospitals and social institutions.”
Yes	R_3NX mQbf9Tr zNX3U	NA	NA	EURO_730824_1	EURO	School trips abroad are forbidden. Already booked school trips must be cancelled. The foreign language study program is also suspended.
NA			NA	OXCGRT_Hungary_2020311_ school	OXCGRT	On 11 March 2020, Hungary’s government declared a state of emergency and closed university campuses. Archive link doesn't work consistently so including orignal for reference too : UNESCO data confirms a partial closure between March 11 and March 14, before shifting to a complete closure:

Meanwhile, the second observation (unique_id: EURO_730824_1) was found to have already been coded in the CoronaNet dataset; as such the coder marked the overlap_assessment as being ‘Yes’ and copied and pasted the corresponding record in the CoronaNet dataset, R_3NXmQbf9TrzN3XU into the matched_record_id column.

Finally, at the time of writing, the third policy has not been assessed for harmonization yet (unique_id: OXCGRT_Hungary_20200311_school). As such, both the overlap_assessment and harmonize_assessment columns take the value of NA.

Step 5 of manually harmonizing the data is still ongoing. However, based on the close to 80k observations that we have assessed so far, we have found that on average 83% of policies in the external dataset were not previously in the CoronaNet dataset. Table 11 provides a breakdown of the overlap assessment by dataset. Overall, HIT-COVID and CoronaNet have the most amount of overlap at 34% while OxCGRT and CoronaNet have the least amount of overlap at 11%.

**Table 11:** Overlap Assessment by Dataset.

overlap assessment	Total	ACAPS	WHO CDC	CIHI	COVID-AMP	WHO EURO	HIT-COVID	OxCGRT
No	0.83	0.79	0.76	0.84	0.82	0.80	0.65	0.89
Yes	0.17	0.21	0.24	0.16	0.18	0.20	0.34	0.11

This table shows the proportion of data that has been found to overlap with the CoronaNet data for a given dataset, where ‘No’ denotes the proportion of data for which no overlap is found for a given dataset and ‘Yes’ denotes the proportion of data for which an overlap is found. Note that assessments of data overlap is still ongoing and these proportions may change the more data is assessed. See Table 6, Step 5, ‘% Overlap Assessment Completed” for progress made on this front at the time of writing.

Meanwhile Table 12 shows the breakdown to date of the harmonization assessment. Recall, that these assessments are only done for policies that are found to not currently be in the CoronaNet dataset, or in other words, for the 83% of the external data assessed to have an overlap_assessment of ‘No’ to date. The harmonization assessments show that around 45% of the data not currently in the CoronaNet dataset is subsequently recoded into the CoronaNet taxonomy and dataset, with around 9% of requiring either additional research or work to find a new link before this is possible. Meanwhile, 25% of the observations are assessed to be duplicates, 21% are not relevant policies and 10% do not have a recoverable link and thus cannot be substantiated and subsequently recoded.

**Table 12:** Harmonization Assessment by Dataset.

harmonize assessment	Total	ACAPS	WHO CDC	CIHI	COVID-AMP	WHO EURO	HIT-COVID	OxCGRT
Harmonized	0.36	0.41	0.44	0.80	0.50	0.37	0.40	0.28
Harmonized with additional work to find a new link	0.03	0.04	0.06	0.01	0.03	0.04	0.04	0.02
Harmonized with additional original research	0.04	0.04	0.07	0.02	0.06	0.05	0.06	0.03
Harmonized with additional original research AND additional work to find a new link	0.02	0.01	0.03	0.00	0.01	0.03	0.01	0.01
Duplicated policy	0.25	0.22	0.22	0.08	0.24	0.17	0.34	0.30
Not a relevant policy	0.21	0.16	0.10	0.04	0.14	0.13	0.12	0.30
Link dead, no other link found	0.10	0.13	0.08	0.06	0.03	0.21	0.04	0.06

This table shows the harmonization assessments for data which has been found to have no overlap with the CoronaNet data (See the ‘No’ row in Table 11 for more information). See the list in the section above for more detail about each harmonization assessment. Note that assessments of data harmonization is still ongoing and these proportions may change the more data is assessed. See Table 6, Step 5, “% Harmonization Assessment Completed” for progress made on this front at the time of writing.

There is however substantial variation for each assessment across the different datasets. Overall, it appears that data from CIHI is often harmonized without the need for substantial extra work and with relatively low issues with duplicated policies, dead links or irrelevant policies. This may in part be due to the fact that CIHI data focuses on subnational Canadian data and is relatively high quality given that it is collected not by volunteers, but by paid contractors. With regards to duplication, HIT-COVID, and OxCGRT appear to have about the similarly high amounts of duplication while there is comparatively little duplication for WHO EURO and as previously mentioned CIHI. The fact that the rate of duplicate data for OxCGRT data is relatively in line with those found for other datasets also suggests that we did not go overboard with our custom OxCGRT deduplication efforts in Step 3a. Dead links appear to be a particular problem for WHO EURO sources while irrelevant policies appear to be particularly high with regards to OxCGRT data. This is likely largely due to previously mentioned differences in OxCGRT and CoronaNet methodology; while OxCGRT documents policies that have ‘no change,’ CoronaNet does not (see the ‘Step 3a. Deduplication within External Datasets’ section for more details).

We conclude by noting that since the last step in the harmonization of the different taxonomies into CoronaNet taxonomy is manual and requires the enlistment of a substantial labor force, we have made significant investments in training research assistants and providing supportive resources for them to minimize the possibility of systematic coding errors. These include:

- Regular workshops for managers and research assistants about data harmonization. These are mandatory for new research assistants and they receive this training along with the original training that we developed to onboard them into the project^6^.
- The design and diffusion of reference material to the research assistants, such as: manuals, spreadsheets, presentations, info-graphics and videos.
- Monitoring and rectification of inconsistencies identified in both the overlap assessment and harmonization assessment stages of the harmonization process by both managers and automated code. If there is an error in the data harmonization process, it is noted and communicated as feedback to research assistants to rectify before it is accepted as a valid harmonized entry.
- Open communication channels for research assistants to receive asynchronous feedback on questions they may have on the data harmonization process through Slack.

While our harmonization efforts are still ongoing, we hope that the methodology we have outlined here can prove useful to others seeking to harmonize similar data or to evaluate the work of others.

### Supplementary information

Supplementary Information

Supplementary Table 1

## Data Availability

Users interested in which observations in the CoronaNet dataset were harmonized from external datasets can reference either the ‘collab’ or ‘collab_id’ columns in the raw event dataset made publicly available on the CoronaNet github repository here: https://github.com/CoronaNetDataScience/corona_tscs/tree/master/data/CoronaNet. The ‘collab’ variable notes which external dataset, if any, an observations was harmonized from and the ‘collab_id’ variable documents the original unique ID which matches the corresponding observation in the original data. We further provide documentation of our methodology and data in the earlier steps of the data harmonization process in an OpenICPSR COVID-19 Data Repository entitled “CoronaNet COVID-19 Policy Responses: Taxonomy Maps and Data for Data Harmonization.”^26^ Interested users can access the taxonomy maps and the input and output data of these taxonomy maps there. Information on the taxonomy maps is also made available on our project website (https://www.coronanet-project.org/external_data_harmonization.html) and information on the data inputs and outputs can similarly also be found in our project git repository (https://github.com/CoronaNetDataScience/corona_tscs/tree/master/data/collaboration and https://github.com/CoronaNetDataScience/corona_tscs/tree/master/RCode/collaboration)

## Code availability

Please refer to the Supplementary Information, Section 2, in order to access the taxonomy maps made for Step 1 of the data harmonization process. Access to these taxonomy maps is also provided through our website here: https://www.coronanet-project.org/external_data_harmonization.html. Meanwhile, we make the code and data for replicating Steps 2 to 3 available in the following folders in the CoronaNet public git repository: https://github.com/CoronaNetDataScience/corona_tscs/tree/master/RCode/collaborationhttps://github.com/CoronaNetDataScience/corona_tscs/tree/master/data/collaboration.
