# Supplementary material for: Harmonizing government responses to the COVID-19 pandemic
Source: Sci Data. 2024 Feb 14;11:204. doi: 10.1038/s41597-023-02881-x (PMC10867014; doi:10.1038/s41597-023-02881-x)
Supplement: Supplementary file 1 — Supplementary Information [file 41597_2023_2881_MOESM1_ESM.pdf]

Supplementary Information

Contents

1 Coverage of subnational policy-making by country and time coverage **2**

2 Taxonomy Maps **4**

3 Comparison between CoronaNet and WHO PHSM data harmonization efforts **6**

3.1 Comparing the scale of harmonization efforts 6

3.2 Comparing the quality of harmonization efforts 8

3.3 Discussion 10

1 Coverage of subnational policy-making by country and time coverage

The following table provides an overview of subnational coverage of COVID-19 policies based on a review of the datasets covered in Supplementary Table 1. Note the time coverage within a given dataset provides an average date across different subnational regions. For example, while the table notes that CoronaNet provides subnational data for Australia until December 2020, in effect this means that for some subnational regions the time coverage goes beyond December 2020 and for other subnational regions it stops before December 2020, with December 2020 being an approximate average date across Australia.

Table S1 Subnational data coverage of COVID-19 PHSM by dataset and time.

country	dataset (average time coverage within the dataset)
Australia	CoronaNet (December 2020), OxCGRT (December 2022)
Brazil	CoronaNet (December 2020), OxCGRT (December 2022)
Canada	CIHI, CoronaNet (December 2020), OxCGRT (December 2022)
China	CoronaNet (January 2021), OxCGRT (January 2023)
France	CoronaNet (May 2021)
Germany	CoronaNet (April 2021)
India	CoronaNet (January 2021), HIT-COVID (December 2020), OxCGRT (December 2022)
Italy	CoronaNet (March 2021)
Japan	CoronaNet (January 2021)
Kazakhstan	CoronaNet (February 2021)
Nigeria	CoronaNet (January 2021)
Switzerland	CoronaNet (January 2021)
Spain	CoronaNet (May 2021)
Russia	CoronaNet (April 2021)
United Kingdom	COVID AMP (December 2020), OxCGRT (December 2022), HealthUK[1] (December 2020)
United States	CoronaNet (December 2020), COVID-19 US State Policies (CUSP) (mid 2021) [2], COVID-19 State Policy Tracker[3] (August 2021), COVID AMP (July 2022), HIT-COVID (November 2020), OxCGRT (December 2022), State Policy Responses to COVID-19 (SPRC19)[4] (April 2020), Yale SOM-Tobin Center State and Local COVID Restriction Database[5] (2021; data not publicly available so this is an approximation)

2 Taxonomy Maps

As we discuss in the Methodology section of our paper, the first step that we took to harmonize data external to the CoronaNet dataset into the CoronaNet taxonomy was to create taxonomy maps between each external dataset and CoronaNet in the R programming language.

While each taxonomy map is necessarily highly specific to the particular mapping needed, they generally follow the same format. That is:

1. The data and necessary packages for mapping the data are loaded into R.
2. Direct matches between variable fields are made where possible.
3. Adjustments to names in administrative fields (e.g. countries, provinces) are made.
4. Mapping between fields which capture information on the content of COVID-19 policies are made. When necessary and possible, adjustments are made to account for the differences in what the mapping should be like in theory and what they were in practice (i.e. for unclean data).
5. The final map is compiled and written into a .csv and .rds file.

Note that additional processing was needed for the OxCGRT data. Because their raw data format is in panel form, while the raw form of the CoronaNet data is in event data form, we needed to developed an additional procedure to extract, collapse and aggregate the OxCGRT panel data into event data form.

Depending on the external dataset, other fields may also be mapped (e.g. de jure compliance with a policy or the demographic target of a policy). However this was not always possible either because the external dataset did not always capture such fields or because it would have been too complex to map. Note because the external data is manually harmonized in Step 5 of the data harmonization process, such fields are ultimately recoded manually into the CoronaNet taxonomy. For the same reason, we did not attempt to make the taxonomy mappings perfectly match for every observation, but rather aimed to create a mapping that would have the highest accuracy for the most number of observations as efficiently as possible

To access these taxonomy maps, please see the following markdown files available in the following links:

- ACAPS Government Measures (ACAPS) - CoronaNet Research Project taxonomy map: https://www.coronanet-project.org/acaps_coronanet_taxonomy
- Canadian Dataset of COVID-19 Interventions (CIHI) - CoronaNet Research Project taxonomy map: https://www.coronanet-project.org/cihi_coronanet_taxonomy

- COVID Analysis and Mapping of Policies (COVID AMP) - CoronaNet Research Project taxonomy map: https://www.coronanet-project.org/COVIDAMP_coronanet_taxonomy
- Johns Hopkins Health Intervention Tracking for COVID-19 (HIT-COVID) - CoronaNet Research Project taxonomy map: https://www.coronanet-project.org/hit_covid_taxonomy
- Oxford COVID-19 Government Response Tracker (OxCGRT) - CoronaNet Research Project taxonomy map: https://www.coronanet-project.org/oxcgrt_coronanet_taxonomy
- WHO Public Health and Safety Measures (WHO PHSM) - CoronaNet Research Project taxonomy map https://www.coronanet-project.org/who_coronanet_taxonomy

The raw data for each external dataset as well as the versions of the data mapped into the CoronaNet taxonomy are available at the CoronaNet git repository here: https://github.com/CoronaNetDataScience/corona_tscs/tree/master/data/collaboration

The same information above is also provided in an OpenICPSR COVID-19 Data Repository entitled "CoronaNet COVID-19 Policy Responses: Taxonomy Maps and Data for Data Harmonization."[6] Interested users can also access the taxonomy maps and the input and output data of these taxonomy maps there.

3 Comparison between CoronaNet and WHO PHSM data harmonization efforts

We are aware of at least one other effort to harmonize PHSM data from different datasets: the World Health Organization's (WHO) PHSM dataset. The World Health Organization's (WHO) PHSM dataset was first published in the summer of 2020 and harmonizes data from five projects: OxCGRT, ACAPS, HIT-COVID, WHO EURO and CDC. Aside from the fact the WHO does not include data from CoronaNet, COVID AMP or CIHI, a crucial difference between our data harmonization efforts and the WHO effort is that the WHO PHSM dataset does not collect original data policies but rather focuses on merging different data sources. Having mapped and evaluated the quality of the WHO PHSM dataset as part of our own data harmonization exercise, we argue that our data harmonization effort improves on their efforts in several respects with regards to the scale and quality of the resulting harmonized data.

The obvious benefit of the WHO PHSM harmonization effort over ours is (i) that they have harmonized data past September 2021 and (ii) that they had been making release weekly updates which harmonizes the latest observations from each underlying dataset. Since August 2022 these weekly updates have stopped however and on their website they report that they have concluded their harmonization exercise. Despite these advantages in time coverage, we argue their approach had come at a substantial cost to data quality. We contend that combining CoronaNet's general methodology of (i) concentrating on a more limited time period and smaller set of countries through to September 2021 (ii) recruiting volunteers all around the world dedicated towards documenting policies for a given country and (iii) using a survey instrument to collect policies [7] and (iv) following a manual data harmonization effort has allowed us to create a more standardized, coherent and valid, dataset compared to the WHO effort. We elaborate on both how our data harmonization efforts compare in terms of scale and quality in the following sections.

Please note that in contrast to our analysis of the subset of the WHO data that we harmonized and discuss in the Methodology section of the paper which was limited to data harmonized before September 21, 2021, in our comparison below we assess the differences between our harmonization efforts and their latest harmonized data, which contains data until August 2022.

3.1 Comparing the scale of harmonization efforts

Overall, we argue that the CoronaNet ongoing harmonization efforts have lead to a dataset that is more compact, insofar as it limits itself to policies made before September 21, 2021, but as such more complete and high quality, than the WHO PHSM harmonization effort, which has harmonized data until August 2022.

We start with a broad comparison of the two harmonization efforts by volume of policies documented. We note that the latest, and final version of the PHSM dataset (dating to August 2022) contains around 121,000 policies,

which is close to 60k policies less than the size of the existing CoronaNet dataset, which at the time of writing documents more than 180,000 policies. On the basis of the number of policies alone, our ongoing harmonization efforts, almost certainly yields a dataset that is more complete than the WHO effort for the time period up until September 2021. By comparison, for this same time period, the WHO PHSM dataset documents close to 96k policies.

Indeed, when we breakdown our harmonization efforts by dataset, we can infer that the WHO PHSM data has less complete data coverage than CoronaNet in part because it does not harmonize data from CoronaNet, COVID AMP or CIHI. Meanwhile, figure S1 further allows us to break down the amount of data in the harmonized WHO PHSM data by dataset and finer slices of time. As it shows, over time, it has come to increasingly rely on data from OXCGT and WHO EURO datasets, as ACAPS, HIT-COVID and CDC stopped data collection.

To take a closer look at how the two data harmonization efforts compare with regards to coverage over time, although the WHO PHSM dataset has indeed been able to harmonize data past September 2021, we believe that this has come at the cost of overall data completeness and quality. That is, given that the pandemic was very much still in full swing from September 2021 to August 2022, with most countries focusing on COVID-19 vaccination in particular, we believe that the 22k+ observations that the WHO PHSM dataset has been able to harmonize from September 2021 to August 2022 can present only a very incomplete picture of the pandemic. Indeed, on further observation, we find that these 22k+ documents policies for 186 countries, with a mean of 120 policies per country. By comparison, the WHO PHSM dataset documented around 44k policies for the same time period one year before, that is, from September 2020 to August 2021 for 228 countries, with a mean of 186 policies per country. For further comparison, we can look at numbers from the CoronaNet dataset from September 2020 to August 2021. Here we find that CoronaNet documented data for 197 countries, with a mean of 347 policies per country. These numbers suggest that by focusing on a more limited period of time, the CoronaNet data harmonization effort is arguably able to build a more coherent dataset for a given time period.

With regards to geographical coverage, though the PHSM dataset provides coverage of 233 regions while our data harmonization efforts only cover 201, these additional covered regions exclusively consist of small island nations or overseas territories which are on average, undercoded within the WHO PHSM dataset[1]. Meanwhile, the WHO PHSM data harmonization effort puts relatively little emphasis on harmonizing subnational data; around 34% of

[1]In the WHO PHSM dataset, there are on average 59 policies which on average covers policies made until mid August 2020 for the following 38 islands and overseas territories and which are not covered in our data harmonization efforts: American Samoa, Anguilla, Aruba, Bermuda, Bonaire, British Virgin Islands, Cayman Islands, Cook Islands, Curacao, Falkland Islands (Malvinas), Faroe Islands, French Guiana, French Polynesia, Gibraltar, Greenland, Guadeloupe, Guam, Guernsey, Isle Of Man, Jersey, Martinique, Mayotte, Montserrat, New Caledonia, Niue, Northern Mariana Islands, Commonwealth Of The, Pitcairn Islands, Puerto Rico, Reunion, Saba, Saint Barthelemy, Saint Helena, Saint Martin, Saint Pierre and Miquelon, Sint Eustatius, Sint Maarten, Turks And Caicos Islands, United States Virgin Islands, Wallis And Futuna

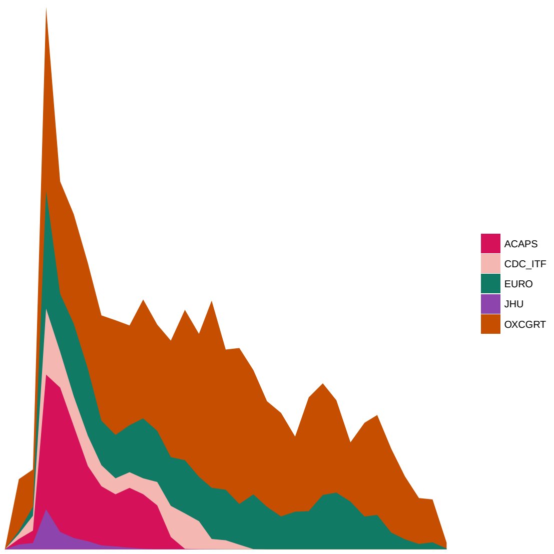

Fig. S1 Number of policies by tracker overtime in the WHO PHSM dataset]

the data it harmonizes is at the sub-national level, compared to 51% for our data harmonization efforts. This is all the more important given that there is substantial subnational variation in the policy making process for many countries, which we discuss in greater detail in the next section.

3.2 Comparing the quality of harmonization efforts

Overall, we have found that the WHO PHSM harmonization efforts suffers from significant problems with regards to data standardization, data coherence as well as source data compared to the Coronanet efforts.

With regards to data standardization, we have identified a number of inconsistencies in the WHO PHSM dataset which makes it difficult to use their data without additional processing. For example, while the WHO dataset captures rich information on the targets of its compiled data in its 'targeted' variable, the usefulness of this variable for analysis is diminished by the fact that it contains more than 13,390 unique entries.[2] While the CoronaNet dataset captures much of this same information, it organizes the information into different fields in more manageable numbers of categories within each, which facilitates a researcher's ability to quantitatively or qualitatively compare different observations. For instance, while the WHO's 'targeted' variable includes entries

[2]Upon closer examination, by simply performing some simple automated cleaning procedures on these categories like removing special characters and making all characters lower case, the actual number of unique entries is closer to 5,900. However even after performing this procedure, the point about lack of standardization still stands. E.g. various observations read: 'al schools', 'all school'; 'all schools' when it would be more useful to use one standard phrasing to refer to all schools.

as varied as ‘secondary schools’, ‘citizens’, and ‘All flights’, CoronaNet documents this information in separate fields (‘secondary schools’ can be found in the ‘type_sub_cat’ variable, which generally captures information on policy sub types. Meanwhile ‘citizens’ can be found in the [target_who_what] variable which captures information on demographic targets, and ‘All flights’ can be found in the [travel_mechanism] variable which generally captures information as to the mode of travel that is restricted). Moreover, though the WHO notes that they standardize names for ’country, territory or area’ in their dataset downloaded, we have consistently found that data on subnational geographic areas are inconsistently documented (documented in their ’area_covered’ variable). For instance, the province of Jammu and Kashmir of India is alternatively coded as ‘jammu and kashmir’, ‘Jammu and kashmir’ or ‘Jammu and Kahsmir’ in the WHO dataset. Because CoronaNet uses a survey instrument to document this data, problems with typos which can make standardization difficult to achieve are avoided.

Additionally, we have found a substantial degree of policy incoherence in the WHO PHSM dataset, both in terms of the quality of the observations harmonized in the dataset as well as in terms of observations not included in the data. With regards to the former, as of August 2022, the WHO PHSM dataset has 890 observations which lack a textual description and 2,911 without a start date. These issues are not present in the CoronaNet data collection methodology because these policy dimensions are collected as forced responses in our survey collection instrument. Meanwhile, with regards to the latter, we have found that there is still a great deal of incoherence in the external data when one simply compiles data from different datasets without doing additional research to fill in the blanks. For instance, while our data harmonization efforts of 7 different datasets have identified 844 external policies for Romania, we found that there were were a substantial number of policies that were not captured by any external data. For instance, even though we identified more 40 policies in the external dataset which could be considered as having the policy type ‘Lockdown’ in the CoronaNet taxonomy, further investigation revealed more than 400 such lockdown policies in Romania because of the government’s strategy of implementing lockdowns in different geographical regions over time. Because CoronaNet also engages in original data collection, such policy gaps can be filled in in conjunction with our data harmonization efforts, although not in the WHO data harmonization efforts. These observations match with our experience that PHSM policies can be very complex and require a) experts who can do the research to substantiate the validity of policies coded in external datasets and conduct additional research to assess whether additional policies need to be documented, and b) evaluate and clean existing policies in external datasets in c) a standardized manner.

Finally problems in the WHO PHSM dataset with regards to missing raw sources and lack of transparency around the data generation process hinders the ability to evaluate the validity of the WHO PHSM dataset. In the current WHO PHSM dataset, there are 5700 missing links, 20k+ additional links that

the WHO PHSM have found to be dead, and 25k+ links which the WHO declared as being ‘unknown’ in terms of whether they have live links or not, but for which no follow up sources are provided. In contrast, CoronaNet only includes data points that have a working link or a screenshot of the original PDF source attached. When CoronaNet research assistants encounter missing or dead links as part of the data harmonization process, they are instructed to either attempt to recover active links with the same information (to date, around 4.7% of the harmonized data) or the observation is not included in the dataset (around 10.2% of the external data). Access to the raw sources is paramount for researchers to independently ascertain the validity and reliability of the subsequent data coded. With regards to the WHO PHSM data generation process, the data providers[8] offer only a basic description of how they process the data. Given the issues with data quality outlined above, greater transparency as to what criterion they use to determine that “the clean, verified data is ready to be shared with WHO and other researchers.” would be welcome.

3.3 Discussion

By laying out the contrast between our data harmonization effort and the WHO data harmonization effort, we hope that readers gain not only a deeper appreciation of the complexity of harmonizing PHSM data, but also for the relative merits of our efforts. Given the volume and complexity of PHSM data as well as the reality of limited resources, we believe that our decision to harmonize data for a more limited period of time results in a higher quality, more complete dataset that can provide a more rigorous foundation for research on the COVID-19 pandemic.

References

[1] Dunn, P., Allen, L., Cameron, G., Alderwick, H.: The Health Foundation, COVID-19 policy tracker: A timeline of national policy and health system responses to COVID-19 in England. 2020. Access available at https://covid19.health.org.uk

[2] Skinner, A., Flannery, K., Nocka, K., Bor, J., Dean, L.T., Jay, J., Lipson, S.K., Cole, M.B., Benfer, E.A., Scheckman, R., *et al.*: A database of us state policies to mitigate covid-19 and its economic consequences. BMC Public Health **22**(1), 1–8 (2022)

[3] Fullman, N., Bang-Jensen, B., Reinke, G., Magistro, B., Castellano, R., Erickson, M., Walcott, R., Dapper, C., Amano, K., Wilkerson, J., Adolph, C.: State-level social distancing policies in response to COVID-19 in the US, Version 1.145. https://github.com/COVID19StatePolicy/SocialDistancing (2020)

[4] Boehmke, F.J., Desmarais, B.A., Eastman, A., Grassel, I., Harden, J.J., Harper, S., Kaboli, L., Ko, H., Oster, E., Saunders, T.M.: Sprc19: A database of state policy responses to covid-19 in the united states. Scientific data **10**(1), 526 (2023). https://doi.org/10.7910/DVN/GJAUGE,

[5] Spiegel, M.: Yale SOM-Tobin Center State and Local COVID Restriction Database. https://som.yale.edu/covid-restrictions (2020)

[6] Cheng, C., Messerschmidt, L., Bravo, I., Waldbauer, M., Bhavikatti, R., Schenk, C., Grujic, V., Model, T., Kubinec, R., Barceló, J.: CoronaNet COVID-19 Policy Responses: Taxonomy Maps and Data for Data Harmonization. Available at OpenICPSR COVID-19 Data Repository: https://doi.org/10.3886/E195081V2, Ann Arbor, MI (2023)

[7] Cheng, C., Barceló, J., Hartnett, A.S., Kubinec, R., Messerschmidt, L.: Covid-19 government response event dataset (coronanet v. 1.0). Nature human behaviour **4**(7), 756–768 (2020). https://doi.org/10.5281/zenodo.5201766

[8] WHO: Global dataset of public health and social measures Data harmonization, processing flow, and data dictionaries for Stage 1 and Stage 2 databases). https://cdn.who.int/media/docs/default-source/documents/phsm/phsm---taxonomy_95529eca-9133-42e5-8549-daff3b208e97.zip?sfvrsn=7b98572e_16 (2020)
